# Gut microbiota and macrophage crosstalk: implications for colitis-associated colorectal cancer

**DOI:** 10.3389/fcimb.2026.1778244

**Published:** 2026-02-24

**Authors:** Wen Lyu, Zhe Zhang, Lu Liu, Yaxing Sun, Binbin Wang, Chuan Zhou, Zhanju Liu, Baisui Feng

**Affiliations:** 1 Department of Gastroenterology, The Second Affiliated Hospital of Zhengzhou University, Zhengzhou, China; 2 Center for Inflammatory Bowel Disease Research and Department of Gastroenterology, Affiliated Suzhou Hospital of Nanjing Medical University, Suzhou Municipal Hospital, Gusu School, Nanjing Medical University, Suzhou, China

**Keywords:** colitis-associated colorectal cancer, gut microbiota, macrophage polarization, metabolites, microbiota-immune interactions

## Abstract

Colitis-associated colorectal cancer (CAC) is one of the most severe complications associated with inflammatory bowel disease (IBD). Within the global landscape of cancer epidemiology, colorectal cancer (CRC) ranks among the leading malignancies in terms of both incidence and mortality. CAC, which arises in the context of IBD—including Crohn’s disease and ulcerative colitis—represents a distinct subtype of CRC that is closely linked to chronic intestinal inflammation. The pathogenesis of CAC is driven by the complex intestinal microenvironment, characterized by dynamic interactions among immune cells, epithelial cells, and the gut microbiota. In this setting, macrophages serve as central regulators of intestinal immunity and exhibit significant plasticity in response to microbial stimuli. Under homeostatic conditions, macrophages contribute to tissue integrity through phagocytic activity and the production of anti-inflammatory mediators. However, during prolonged inflammation, persistent exposure to a dysbiotic microbiota induces functional reprogramming of macrophages toward a pro-tumorigenic phenotype. Pathogenic bacteria enriched in CAC tissues—such as *Fusobacterium nucleatum* and *Escherichia coli*—secrete virulence factors that promote macrophage polarization into an immunosuppressive M2-like state, thereby facilitating the establishment of a tumor-permissive microenvironment. In contrast, beneficial bacterial genera such as *Bifidobacterium* and *Lactobacillus* are frequently depleted in CAC, compromising the capacity of macrophages to mediate anti-inflammatory and immunoregulatory responses and further accelerating disease progression. This article reviews the intricate bidirectional interactions between the gut microbiota and macrophages, and emphasizes the molecular mechanisms by which gut microbes influence macrophage polarization and function, as well as how the phenotypic changes of macrophages shape the tumor microenvironment to promote the development of CAC. Additionally, the article explores potential therapeutic strategies aimed at inhibiting the inflammation-to-cancer transition by targeting these interactions.

## 1 Introduction

Colitis-associated colorectal cancer (CAC) is a distinct type of colorectal cancer (CRC) that develops from persistent colitis in patients with inflammatory bowel disease (IBD), including ulcerative colitis (UC) and Crohn’s disease (CD). Its pathological evolution begins with non-neoplastic inflammatory epithelium and ultimately progresses to cancer (Wu et al., 2018). Compared with sporadic CRC, the distinctive features of CAC include the presence of multiple lesions, more severe pathological types, and a poorer prognosis (Zhang M. et al., 2023). The significant characteristics of CAC encompass genetic instability, such as chromosomal instability, microsatellite instability, hypermethylation, and alterations in non-coding RNAs (Yin et al., 2023). CAC is not only closely associated with chronic or dysregulated inflammation (Feng et al., 2020), but also strongly linked to dysbiosis resulting from alterations in the composition of the gut microbiota (Kwong et al., 2018; Sakai et al., 2021). In the inflammatory intestinal environment, opportunistic pathogens such as *Escherichia coli* and their metabolites persistently activate immature colonic macrophages, thereby further exacerbating chronic inflammation and promoting tumorigenesis (Yang Y. et al., 2020), Consequently, modulating the intestinal inflammatory response has emerged as an effective strategy for the prevention of CAC (Baars et al., 2011; Lu et al., 2018).

Macrophages serve as crucial regulators of intestinal immune homeostasis and primarily modulate the progression from IBD to colorectal cancer through cytokine secretion (Jin et al., 2024). During the early inflammatory phase of CAC, macrophages predominantly exhibit an M1-like pro-inflammatory phenotype. A large number of infiltrating M1 macrophages exacerbate the mucosal damage caused by inflammation and promote the transformation from inflammation to tumorigenesis. In contrast, during the middle and advanced stages of CAC, M2-like macrophages become the dominant subset and exert tumor-promoting functions. M2 macrophages typically facilitate cancer cell metastasis, angiogenesis, and proliferation through various anti-inflammatory mechanisms (McLean et al., 2011; Boutilier and Elsawa, 2021). Furthermore, macrophages contribute to the initiation and progression of CAC through the production of reactive oxygen species (ROS), nitric oxide synthase (NOS), and pro-inflammatory cytokines (Grivennikov et al., 2009).

The gut microbiota is established at birth and undergoes dynamic changes throughout life while maintaining a symbiotic relationship with the host, serving as an integral component of human physiology (Su et al., 2023). The gut microbiota participates in the synthesis and metabolism of various intestinal substances, including energy homeostasis, glucose metabolism, and lipid metabolism (Sonnenburg and Bäckhed, 2016), Its metabolites, in conjunction with gut microbiota, contribute to the regulation of the intestinal microecology (Yin et al., 2023). The role of altered gut microbiota in diseases is associated with gut microbiota metabolites, such as metabolites like short-chain fatty acids (SCFAs) and bile acids (BAs) from different microorganisms (Su et al., 2023). These metabolites contribute to the promotion of gut barrier maturation and immune homeostasis (Hu et al., 2022). Alterations in the gut microbiota contribute to the occurrence and development of CAC and other gastrointestinal diseases (Lloyd-Price et al., 2019; Dixit et al., 2021; Yin et al., 2023). Gut microbiota metabolites play a crucial role in maintaining systemic and intestinal homeostasis by suppressing pro-inflammatory immune cells and promoting the differentiation and function of immunosuppressive cells (Su et al., 2023). Specifically, Butyrate, a SCFAs, inhibits intestinal inflammation and enhances the intestinal microenvironment by suppressing M1 macrophage polarization (Shao et al., 2021). The tryptophan (Trp) metabolite Indole-3-lactic acid (ILA) interferes with pro-inflammatory macrophage differentiation and alleviates the inflammatory response through activation of the PI3K/AKT signaling pathway (Li et al., 2024). Under high-fat diet (HFD) conditions, the secondary bile acid Deoxycholic acid (DCA) promotes pyroptosis mediated by Gasdermin D in macrophages and enhances IL-1β secretion, thereby exacerbating colitis (Huang et al., 2023). The gut microbiota and its metabolites regulate macrophage polarization, further modulating the gut microenvironment and contributing to the transformation of the inflammatory microenvironment into a pro-tumorigenic environment.

## 2 Driving mechanisms of gut dysbiosis and metabolite imbalance

### 2.1 Characteristics and carcinogenic mechanisms of gut dysbiosis

Gut microbiota contributes to pathogen resistance and helps maintain the integrity of the mucosal barrier (Chandrasekaran et al., 2024). Gut dysbiosis in IBD patients, characterized by reduced microbial diversity and an imbalance between commensal and pathogenic bacteria. This dysbiosis compromises the intestinal barrier, resulting in increased intestinal permeability and exacerbated inflammation (Ren et al., 2025). Dysbiosis leads to an imbalance in microbial metabolites, characterized by a decrease in protective metabolites and an accumulation of pathogenic metabolites. The combined effect of these two types of changes jointly results in the disruption of the intestinal mucosal barrier and dysregulation of immune inflammation. The gut microbiota constitutes a complex ecosystem in which gut bacteria are primarily categorized into three groups (**Table 1**): (1) protective commensal bacteria, such as *Bifidobacterium* and *Lactobacillus*; (2) pathogenic bacteria, such as *Fusobacterium nucleatum* (*F. nucleatum*) and *enterotoxigenic Bacteroides fragilis* (ETBF); (3) conditionally pathogenic bacteria, such as *Escherichia coli* (*E. coli*).

**Table 1:** Gut microbiota and CAC.

Microbiota	Effect	Reference
*Fusobacterium nucleatum*	*Fusobacterium nucleatum* promotes M1 macrophage polarization, and modulating the tumor microenvironment.	(Kostic et al., 2013; Liu L. et al., 2019)
*Enterotoxigenic* *Bacteroides fragilis*	ETBF promoted the M2 polarization of macrophages to promote tumor growth, resulting in the occurrence and progression of CRC.	(Chai et al., 2021)
pks^+^*Escherichia coli* (Eg: *E. coli* NC101)	Creates a host microenvironment that promotes DNA damage and tumorigenesis.	(Arthur et al., 2012)
*Lactobacillus rhamnosus*	Inhibits macrophage M1/M2 and alleviate inflammation and inhibit carcinogenic signals in an AOM/DSS mouse model.	(Xu et al., 2021)
*Bifidobacterium breve*	Metabolic generation of ILA prevents tumorigenesis by regulating macrophage differentiation in CAC mice.	(Li et al., 2024)

#### 2.1.1 Pathogen enrichment

*F. nucleatum* is an invasive, adherent, and pro-inflammatory anaerobic bacterium that accumulates in the intestines of patients with IBD or CRC and exhibits carcinogenic properties (Kostic et al., 2013). Research indicates that *F. nucleatum* exacerbates colitis by disrupting epithelial integrity and modulating M1 macrophage polarization (Liu L. et al., 2019). ETBF secretes the pathogenic Bacteroides fragilis toxin, which binds to specific receptors on colonic epithelial cells and activates the Wnt and nuclear factor-kappa B (NF-κB) signaling pathways. This activation leads to increased epithelial cell proliferation, release of pro-inflammatory mediators, and DNA damage. Furthermore, it disrupts the intestinal barrier, thereby promoting tumorigenesis (Boleij et al., 2015; Hwang et al., 2020). In the inflammatory intestinal environment, *E. coli* and its metabolites continuously activate immature colonic macrophages, further exacerbating chronic inflammation and promoting tumorigenesis (Yang Y. et al., 2020). The detection rate of *E. coli* harboring the polyketide synthase (*pks*) genomic island is significantly higher in the tissues of patients with IBD and CRC than in healthy individuals, suggesting a potential role in the development of intestinal lesions (Arthur et al., 2012). *pks^+^ E. coli* can cause DNA damage in host epithelial cells (Cuevas-Ramos et al., 2010), and induce functional mutations associated with the p53 and Wnt signaling pathways when human colon organoids are acutely exposed to *pks^+^ E. coli*, thereby increasing the risk of CRC (Iftekhar et al., 2021). In summary, pathogenic bacteria such as *F. nucleatum*, ETBF, and *pks^+^ E.coli* collectively represent a key link in dysbiosis-driven carcinogenesis by directly damaging the epithelium, activating oncogenic signaling pathways, and inducing DNA damage.

#### 2.1.2 Protective commensal depletion

Protective commensal bacteria play a crucial role in modulating the composition of the gut microbiota and inhibiting the colonization of gut pathogens, thereby contributing to the establishment of a healthy intestinal mucosa (Kumar et al., 2020). Common protective commensal bacteria include *Lactobacillus* and *Bifidobacterium* (Kerry et al., 2018). The genera *Bifidobacterium* and *Lactobacillus* can exert various effects on the host, including competitive exclusion of pathogens, restoration of microbial homeostasis, regulation of intestinal transit time and SCFAs production, and enhancement of mucosal barrier function (Hill et al., 2014). Their reduction in patients with CAC weakens the protective effect of the gut microbiota on the intestine.

*Lactobacillus* is a genus of Gram-positive anaerobic bacteria, which exerts anti-inflammatory and immunomodulatory effects through multiple mechanisms. Intestinal *lactobacillus* stimulate lactic acid production and activate hypoxia-inducible factor (HIF)-2α-mediated signaling, thereby promoting intestinal health (Shao et al., 2022). *Lactobacillus plantarum* can stimulate the production of IL-10, increase the proportion of regulatory T cells (Tregs), and improve the imbalance between Th17 and Treg cells in the intestines of IBD patients, exerting anti-inflammatory effects (Le and Yang, 2018). In mouse models, *Lactobacillus reuteri* not only inhibits the polarization of M1-like macrophages and promotes the M2-like phenotype, but also suppresses neutrophil recruitment and dendritic cell expansion in the intestinal mucosa, and increases the frequency of Tregs in mesenteric lymph nodes, thereby alleviating inflammatory stress in mice (Dias et al., 2021; Liu HY. et al., 2022). *Lactobacillus rhamnosus* also possesses the ability to modulate the relative abundance of immune cells. It reduces the Th17/Treg ratio via the JAK-STAT signaling pathway, indicating its significant value in preventing excessive inflammatory activation (Chiu et al., 2010), In the CAC mouse model, administration of *Lactobacillus rhamnosus* alleviates inflammation, reduces tumor number and average size, and improve M1 macrophage polarization and fibrosis. Moreover, while suppressing these inflammatory responses, the phosphorylation of oncogenic signals Akt and STAT3 is also inhibited (Xu et al., 2021). It can be seen that lactic acid bacteria interfere with intestinal and systemic inflammatory responses from multiple directions and perspectives by altering the quantity, recruitment, and differentiation of immune cells, thereby limiting the progression and exacerbation of IBD and its associated cancers.

*Bifidobacterium* is among the first microbial colonizers of the human and animal intestine. Of these species, *Bifidobacterium longum* is particularly prevalent in the intestines of both infants and adults (O’Callaghan and van Sinderen, 2016; Bottacini et al., 2017). Compared with healthy individuals, the abundance of *Bifidobacterium* and *Bifidobacterium longum* is significantly reduced in the fecal samples of patients with intestinal diseases (Arboleya et al., 2016; Li et al., 2024). *Bifidobacterium* exerts anti-inflammatory effects in murine colitis by promoting the production of SCFAs (Singh et al., 2020). It enhances aryl hydrocarbon receptor (AhR) activity by increasing the levels of endogenous AhR ligands, thereby contributing to anti-inflammatory responses (Cui et al., 2022). Furthermore, *Bifidobacterium* increases the frequency of mucosal Tregs, leading to amelioration of intestinal inflammation (Mańkowska-Wierzbicka et al., 2020). The protective effects of *Bifidobacterium longum* on intestinal epithelial cells involve multiple mechanisms (Yao et al., 2021): reducing myeloperoxidase activity and ROS production to mitigate oxidative stress (Abrantes et al., 2020; Wang et al., 2021); downregulating the expression of pro-inflammatory cytokines such as IL-6, IL-8, and TNF-α, as well as suppressing the NF-κB signaling pathway, thereby modulating intestinal immune responses and preserving epithelial integrity (MacPherson et al., 2017; Choi et al., 2019; Sato et al., 2021); and secreting beneficial metabolites and enhancing intestinal adhesion to inhibit colonization by pathogenic bacteria (Ehrlich et al., 2020; Meng et al., 2020). *Bifidobacterium breve* (*B. breve*)-mediated Trp metabolism ameliorates the precancerous inflammatory intestinal environment by promoting the differentiation of immature colonic macrophages, thereby inhibiting tumorigenesis (Li et al., 2024).

In summary, within the intestinal microenvironment during the initiation and progression of CAC, microbial dysbiosis is reflected not only in alterations in bacterial composition but, more importantly, in the disruption of the microbiota’s overall ecological functions. These dysbiotic microbial communities modulate the intestinal chemical environment through their metabolic activities, thereby rendering microbiota-derived metabolites key drivers in the processes of inflammation and carcinogenesis. In the following section, we focus on selected key microbiota metabolites and systematically discuss their roles in regulating inflammatory and immune responses to influence CAC progression.

### 2.2 Metabolite-mediated bidirectional crosstalk

#### 2.2.1 Tumor-suppressive metabolites

##### 2.2.1.1 Short-chain fatty acids

Short-chain fatty acids, carboxylic acids produced by gut microbiota through the fermentation of dietary fiber in the cecum and colon, play essential roles in human health and disease (Koh et al., 2016; Ratajczak et al., 2019). They are considered potential therapeutic targets for CRC due to their ability to regulate energy metabolism, enhance intestinal barrier integrity, and modulate immune responses (Hou et al., 2022). SCFAs primarily include acetate (C2), propionate (C3), and butyrate (C4). Among them, butyrate participates in multiple physiological functions, including trans-epithelial transport, amelioration of mucosal inflammation, alleviation of oxidative stress, enhancement of epithelial barrier integrity, and prevention of CRC (Hamer et al., 2008). Furthermore, butyrate serves as a primary energy source for colonic epithelial cells (Schoeler and Caesar, 2019). Patients with IBD commonly exhibit an imbalance in the gut microbiota. The marked reduction of butyrate-producing bacteria may reflect the role of butyrate in maintaining intestinal homeostasis through the regulation of gut macrophage function (Dong et al., 2022). Butyrate reprograms macrophage metabolism toward oxidative phosphorylation and induces an anti-inflammatory tolerant phenotype (Scott et al., 2018). It negatively regulates the NLRP3-mediated inflammatory signaling pathway, suppresses M1 macrophage activation and the secretion of pro-inflammatory mediators such as IL-18 and IL-1β, reduces intestinal inflammation, improves the intestinal microenvironment, and thereby inhibits the initiation and progression of CRC (Shao et al., 2021). Additionally, butyrate induces apoptosis in colon cells by promoting ROS generation and the release of pro-apoptotic factors, contributing to CRC suppression (Vernia et al., 2021). Butyrate-mediated histone deacetylase (HDAC) inhibition also blocks the activation of Akt and ERK1/2, which are essential for colorectal cancer cell migration and invasion (Li et al., 2017).

##### 2.2.1.2 Indole-3-lactic acid

Indole-3-lactic acid, a Trp metabolite, is produced by various *Bifidobacterium* species through metabolic processes (Fang et al., 2022; Li et al., 2024). Distinct from *Bifidobacterium* strains associated with adults or animals, *B. breve lw01*, isolated from infant feces, produces relatively high levels of ILA (Li et al., 2024). By activating the AhR in macrophages, it regulates macrophage differentiation (Li et al., 2024) and intestinal mucosal barrier integrity (Scott et al., 2020), thereby suppressing inflammation (Domínguez-Acosta et al., 2018). Moreover, ILA generated through microbial metabolism reduces the proportion of immature colonic macrophages while increasing that of mature colonic macrophages, without altering total macrophage infiltration (Li et al., 2024). *Bifidobacterium longum subsp. infantis* (*B. infantis*) is one of the dominant species in the gut microbiota of breastfed infants and a key member of the infant gut microbiome (LoCascio et al., 2007; Garrido et al., 2016). It exerts anti-inflammatory effects by activating the AhR and nuclear factor erythroid 2-related factor 2 (Nrf2) pathways through ILA production (Ehrlich et al., 2020). Yu K et al. have demonstrated that ILA downregulates the expression of CCL2/7 in epithelial cells by inhibiting glycolysis, NF-κB and HIF signaling pathways, thereby reducing the accumulation of inflammatory macrophages (Yu et al., 2023). Meanwhile, ILA exerts anti-inflammatory effects by activating the AhR and STAT1 pathways in immature epithelial cells stimulated with IL-1β or lipopolysaccharide (LPS) (Meng et al., 2020; Huang et al., 2021). ILA derived from *Lactobacillus plantarum* ameliorates the development of colorectal tumors by enhancing the function of tumor-infiltrating CD8^+^ T cells (Zhang Q. et al., 2023). ILA modulates cellular interactions within the intestine by suppressing crosstalk between intestinal epithelial cells and macrophages, thereby maintaining intestinal homeostasis and serving as a key mediator in preventing inflammation-to-cancer transformation.

#### 2.2.2 Tumor-promoting metabolites

##### 2.2.2.1 Deoxycholic acid

Under HFD conditions, the gut microbiota and its metabolic activities are disrupted. Clostridium converts cholic acid into substantial amounts of DCA via the 7α-dehydroxylation reaction, leading to abnormal accumulation of DCA in the colon. DCA is subsequently reabsorbed into the portal venous system through passive diffusion. The accumulation of DCA has been established as a key initiating factor that drives aberrant activation and inflammation of colonic macrophages, and is closely associated with the pathogenesis of CRC (Ridlon et al., 2006; Cao et al., 2014; Joyce and Gahan, 2016; Zhao et al., 2016). DCA disrupts the integrity of cells and upregulates the expression of pro-inflammatory cytokines, including IL-1β, IL-6, and TNF-α (Liu et al., 2018). Under HFD conditions, the accumulation of DCA resulting from gut microbiota dysbiosis is a key factor triggering M1 polarization and inflammation in colonic macrophages. DCA induces macrophage polarization toward the pro-inflammatory M1 phenotype in a dose-dependent manner. This process does not involve the classic bile acid receptors (TGR5/FXR), but is mediated at least in part by transactivation of TLR2 through the M2 muscarinic acetylcholine receptor (M2-mAChR), which subsequently activates downstream MAPK and NF-κB inflammatory signaling pathways. Furthermore, the DCA-M2-mAChR axis upregulates TLR2 expression via the AP-1 transcription factor, establishing a positive feedback loop that sustains the inflammatory response (Wang L. et al., 2020). This mechanism ensures the sustained maintenance of inflammatory signals, providing a novel molecular basis for explaining the chronicity of colonic inflammation under HFD conditions. In addition, research has shown that DCA can activate the NLRP3 inflammasome in macrophages via the S1PR2-cathepsin B pathway in a dose-dependent manner, promote the secretion of bioactive IL-1β, and thereby exacerbate DSS-induced colitis in mice (Zhao et al., 2016).

In summary, substantial accumulation of DCA under specific conditions promotes the progression of colitis. These mechanisms collectively indicate that DCA is a significant tumor-promoting factor. Such abnormal DCA accumulation serves as a crucial link between dysbiosis, M1 macrophage-driven chronic inflammation, and inflammation-to-cancer transformation.

##### 2.2.2.2 Lipopolysaccharide

Lipopolysaccharide, a member of the gut microbial metabolite family, is a major component of the outer membrane of Gram-negative bacteria and a highly inflammatory endotoxin (Gandhi, 2020). LPS itself is insufficient for effective innate immune activation. Therefore, it requires lipopolysaccharide-binding protein (LBP) to form a high-affinity complex with its lipid A moiety, facilitating transfer to CD14. This enables the translocation of LPS to the endotoxin receptor complex composed of toll-like receptor 4 (TLR4) and MD2 (Bode et al., 2012). Similar to other members of the TLR family, TLR4 is a type I transmembrane protein featuring an extracellular domain containing leucine-rich repeat (LRR) sequences and an intracellular Toll/IL-1 receptor (TIR) domain, serving as the critical sensor for LPS (Rossol et al., 2011). MD2 associates with the extracellular domain of TLR4, and is essential for the cell surface expression of TLR4, the recognition of LPS, and ligand-induced receptor clustering (Nagai et al., 2002; Kobayashi et al., 2006). TLR4 initiates a signaling cascade by recruiting adaptor proteins such as myeloid differentiation primary response 88 (MyD88) and TIR-domain-containing adaptor-inducing interferon-β (TRIF), leading to the activation of NF-κB and phosphorylation of interferon regulatory factor 3 (IRF3).This process enhances the production of pro-inflammatory cytokines, thereby promoting the initiation and progression of CAC (Xu et al., 2024). LPS is a classical signal that induces macrophage polarization toward the M1 phenotype. This process is mediated by TLR4, which activates M1 macrophages, promotes their metabolic shift to glycolysis, and enhances cancer-related inflammation in tumor-associated macrophages (TAMs) (Schmid et al., 2011; Wang et al., 2019). Concurrently, LPS disrupts tight junctions in intestinal epithelial cells by activating the TLR4/NF-κB and JAK/STAT3 signaling pathways, leading to increased intestinal permeability (Wang JW. et al., 2020).

## 3 Dynamic regulation of macrophage polarization and the inflammation-to-cancer transition

Macrophages play diverse roles throughout the progression from colitis to CAC due to their high plasticity. These cells differentiate into M1 and M2 phenotypes, which exert distinct functions in inflammation and tumorigenesis. Prior to tumor formation, M1 macrophages contribute to a pro-inflammatory environment that promotes tumorigenesis. Following CAC development, M1 polarization exerts anti-tumor effects by enhancing tumor immunity, whereas M2 macrophages promote tumor progression and metastasis (Zhang M. et al., 2023).

### 3.1 Dual roles and dynamic transitions in macrophage polarization

As a crucial component of the intestinal immune system, macrophages are widely distributed throughout the body and exhibit high plasticity. They play a central role in maintaining immune homeostasis and host defense through multiple mechanisms, including pathogen phagocytosis and clearance, antigen presentation, initiation of inflammatory responses, and cytokine secretion. Moreover, their capacity for phenotypic switching is essential for coordinating tissue maintenance, repair, and remodeling (Sica and Mantovani, 2012; Wang L. et al., 2020; Liu L. et al., 2022).

Macrophage polarization refers to the process by which macrophages acquire distinct phenotypic and functional characteristics in response to microenvironmental stimuli and signals present in specific tissues (Sica and Mantovani, 2012). According to distinct activation modes, macrophages adopt a pro-inflammatory M1 phenotype (classical activation) or an anti-inflammatory M2 phenotype (alternative activation) (Italiani and Boraschi, 2014). The emergence of these phenotypes results from macrophage polarization in response to specific signals within the tissue microenvironment (Martinez and Gordon, 2014; Liu L. et al., 2022; Deng et al., 2025). In infected tissues, macrophages are initially polarized toward the pro-inflammatory M1 phenotype, which secretes inflammatory cytokines to enhance the inflammatory response and assist the host in combating pathogens. Subsequently, macrophages undergo polarization toward the anti-inflammatory M2 phenotype to promote tissue repair (Yunna et al., 2020). Interestingly, in tumor tissues, M1 macrophages exert anti-cancer effects by enhancing tumor immunity, whereas M2 macrophages are known to suppress antitumor immune responses (Zhang M. et al., 2023). This indicates that macrophage polarization can influence not only the development of CAC through modulation of inflammation but also directly impact CAC by shaping the tumor microenvironment (Zhang M. et al., 2023). In addition to massive infiltration and disruption of polarization balance, macrophages contribute to CAC progression through other mechanisms, including regulation of inflammatory cytokine and tumor-promoting factor secretion, as well as targeting the NF-κB signaling pathway within macrophages.

#### 3.1.1 M1 macrophages: a dual role in pro-inflammatory response and tumor surveillance in colitis-cancer transformation

When resting macrophages (M0) are exposed to inflammatory stimuli such as LPS, IFN-α, IL-12, and IL-23, they polarize toward the pro-inflammatory M1 macrophages (Luo et al., 2024). Each macrophage subtype exhibits distinct surface biomarkers. The primary surface markers of M1 macrophages include CD80, CD86, and TLR-4 (Orecchioni et al., 2019). These cells secrete a range of pro-inflammatory cytokines and chemokines, such as TNF-α, IL-6, IL-1β, ROS, CXCL9, CXCL10, CXCL11, CCL2, CCL3 and others (Wu et al., 2020). Polarized M1 macrophages possess enhanced antigen-presenting capacity. Consequently, they exhibit potent pro-inflammatory, antibacterial, and antitumor activities (Zhang M. et al., 2023). The inactivation of these inflammatory mediators leads to the resolution of inflammation, causing pro-inflammatory M1 macrophages to undergo apoptosis or transform into anti-inflammatory M2 macrophages (Murray and Wynn, 2011). In tumor tissues, polarized M1 macrophages promote tumor immunity and alleviate the tumor burden (Chen et al., 2021; Natoli et al., 2021). M1 macrophages enhance the activity of immune cells, such as natural killer (NK) cells and CD8^+^ T cells, by producing a range of pro-inflammatory mediators, including TNF-α and IL-6, thereby augmenting their capacity to target and eliminate tumor cells. Furthermore, M1 macrophages directly induce tumor cell death and suppress tumor growth through the generation of ROS (Aminin and Wang, 2021; Zhang M. et al., 2023). Macrophage scavenger receptor A1 (SR-A1) is a pattern recognition receptor predominantly expressed in macrophages. In macrophages, SR-A1 suppresses both classical and non-classical activation of the NF-κB signaling pathway through TRAF6 and TRAF3, respectively, thereby attenuating the development of colitis and CAC (Dai et al., 2020). M1 macrophages promote the expression of TNF-related apoptosis-inducing ligand in adipose tissue-derived stem cells (ASCs). Subsequently, adipose tissue-derived stem cells induce apoptosis in CD133^+^ tumor cells and reduce M2 macrophage accumulation via TNF-related apoptosis-inducing ligand, contributing to the inhibition of CAC progression (Eom et al., 2020).

#### 3.1.2 M2 macrophages: mediators of immunosuppression and tumor promotion in colitis-cancer transformation

M2 macrophages play a critical role in immune regulation and the maintenance of immunological tolerance. Polarization toward the M2 phenotype is induced by Th2 cytokines, including IL-4, IL-10, and IL-13 (Verreck et al., 2004), These macrophages are characterized by the expression of specific surface markers, such as CD206 and CD163, and the secretion of anti-inflammatory mediators, including Arg-1, IL-10, and TGF-β, which collectively serve to suppress excessive inflammatory responses and facilitate tissue repair (Guo et al., 2013; Tang et al., 2017).M2 macrophages also play a crucial role in tissue repair and anti-parasitic immune responses (Odegaard and Chawla, 2011). Depending on the activating stimuli, M2 macrophages can be further categorized into four distinct subtypes: M2a, M2b, M2c, and M2d (Zhang M. et al., 2023). As a predominant subset of TAMs, M2 macrophages possess potent phagocytic activity, enabling the clearance of cellular debris and apoptotic cells. They also contribute to tissue repair and angiogenesis, suppression of immune responses, and promotion of tumor progression and metastasis (Rao et al., 2022). Intriguingly, several studies have revealed that M2 macrophages not only contribute to tumor progression but also elevate the incidence of CAC. This phenomenon may be associated with the tumor-promoting function of M2 macrophages during the early stages of tumorigenesis (Coburn et al., 2019; Yuan et al., 2021). EGFR signaling is essential for sustaining the growth and facilitating the metastasis of CRC. It has been reported that in macrophages, the EGFR signaling activates M2 polarization via STAT6, thereby promoting angiogenesis and the progression of CAC (Hardbower et al., 2017). The imbalance of ETBF in the intestine stimulates STAT3-mediated M2 macrophage polarization, which promotes the malignant transformation of adenomas and thus facilitates the occurrence and development of CAC (Chai et al., 2021). Additionally, MyD88 signaling in myofibroblasts enhances the secretion of osteopontin, which drives M2 polarization of macrophages via activation of the STAT3 and PPARγ pathways, thus exacerbating colitis-associated tumorigenesis (Yuan et al., 2021).

#### 3.1.3 Beyond the M1/M2: macrophage heterogeneity from a single-cell perspective

With the advancement of single-cell RNA sequencing (ScRNA-seq) technology, the heterogeneity of macrophages has been shown to extend far beyond the classical M1/M2 dichotomy. ScRNA-seq is a powerful technique for dissecting the complexity of solid tumors, enabling high-resolution characterization of cellular diversity and heterogeneous phenotypic states (Papalexi and Satija, 2018; Zhang and Zhang, 2019). Single-cell analysis has further revealed the complexity of TAMs across multiple cancer types. TAMs represent a heterogeneous cell population that contributes to malignancy through the production of tumor-promoting and angiogenic growth factors, extracellular matrix (ECM) remodeling, and induction of immunosuppression (DeNardo and Ruffell, 2019).

Two distinct TAM populations, composed of C1QC^+^ and SPP1^+^ TAMs, have been identified in CRC through ScRNA-seq. Both of these populations may originate from an intermediate state of FCN1^+^ monocyte-like cells within the tumor, and neither aligns with the classical M1 and M2 dichotomous phenotypes (Müller et al., 2017; Azizi et al., 2018; Zhang et al., 2020). C1QC^+^ TAMs preferentially express genes associated with phagocytosis and antigen presentation, whereas SPP1^+^ TAMs are enriched in angiogenesis-regulating factors. Compared to the mucosa of healthy donors, SPP1^+^ TAMs are significantly enriched in tumor tissues, indicating their critical involvement in CRC tumorigenesis. Furthermore, SPP1^+^ TAMs exhibit specific enrichment in signaling pathways related to colorectal adenoma and metastatic liver cancer, suggesting a functional role in promoting tumorigenesis and metastasis in CRC. A gene expression signature characterized by low levels of C1QC^+^ TAM markers and high levels of SPP1^+^ TAM markers is associated with poor prognosis in CRC patients (Zhang et al., 2020).

In addition, CX3CR1^+^ macrophages in the intestinal lamina propria promote intestinal homeostasis through immunomodulatory IL-10. Experimental results indicate that the interaction between CX3CR1 and its ligand CX3CL1/Fractalkine induces upregulation of heme oxygenase-1 (HMOX-1) in macrophages via STAT3 phosphorylation (Marelli et al., 2017). HMOX-1 is a critical anti-inflammatory and antioxidant enzyme expressed by macrophages and other cell types (Guo et al., 2001; Whittle and Varga, 2010), CX3CR1^+^ macrophages play an essential role in controlling intestinal inflammation and preventing tumorigenic chronic colitis through the regulation of HMOX-1 expression (Marelli et al., 2017). Another distinct subtype, S100A9^+^ Mac/Mono cells, promotes the C3 molecular subtype of CRC at the single-cell level. The C3 subtype is characterized by a high abundance of S100A9^+^ macrophages and is associated with poor prognosis. Within the C3 subtype of CRC, S100A9^+^ macrophages demonstrate the capacity to modulate T cell activity through cell-cell contact (Bao et al., 2023).

These findings suggest that a combined strategy targeting specific subtypes of macrophages and immune checkpoints may offer promising prospects for enhancing the efficacy of immunotherapy in certain CRC.

### 3.2 Key pathways through which microbial metabolites regulate macrophage polarization

**Figure 1:**
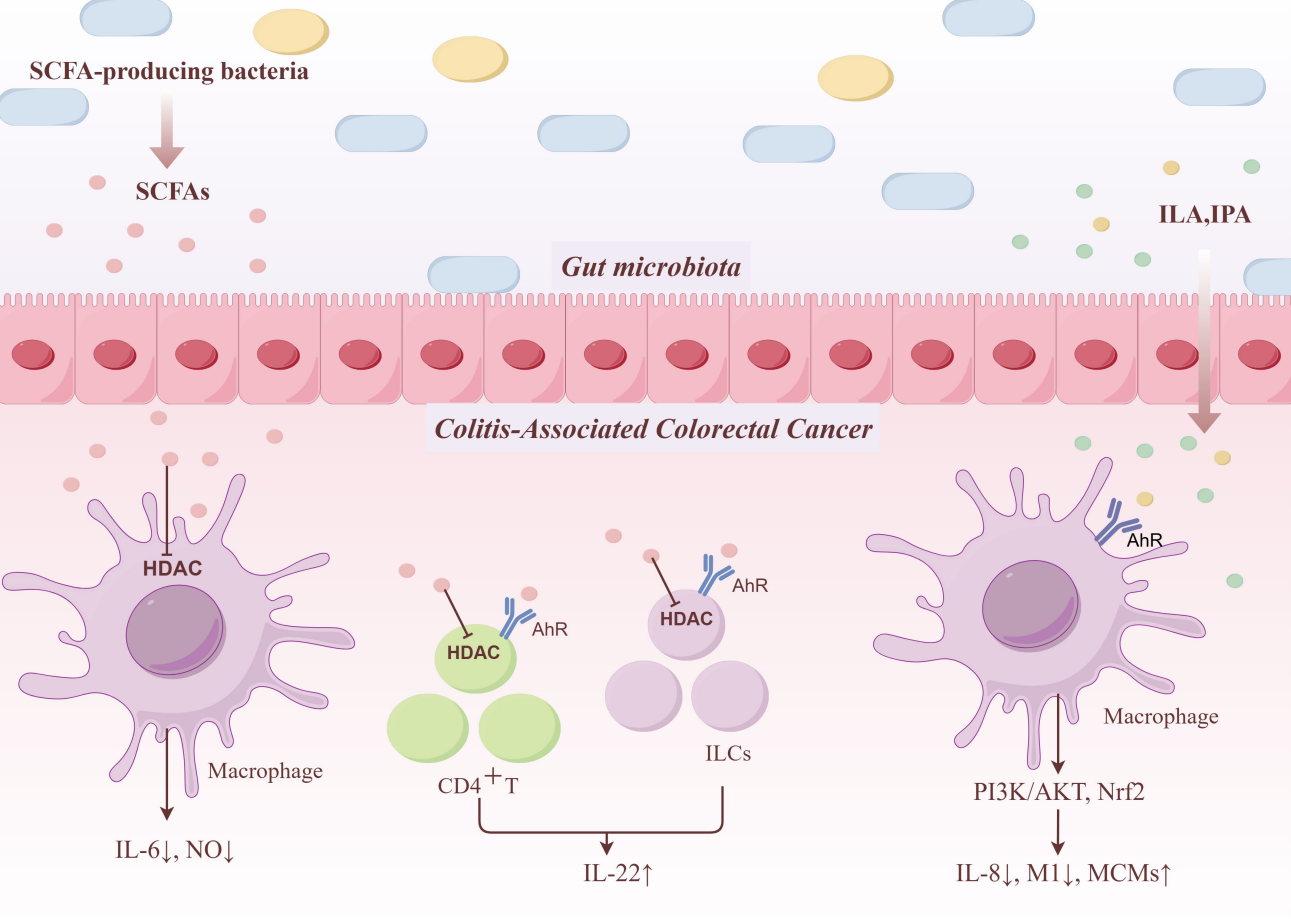
In the gastrointestinal tract, AHR ligands are largely identified as metabolites of tryptophan and indole metabolism, such as ILA and IPA. They reduce the production of the pro-inflammatory cytokine IL-8 in intestinal epithelial cell lines by activating AhR in intestinal epithelial cells and its downstream Nrf2 and PI3K/AKT pathways. Additionally, they decrease the activation of M1 macrophages and increase the proportion of mature macrophages. SCFAs inhibit the secretion of inflammatory factors, such as NO and IL-6, by macrophages through HDAC and promote the production of IL-22 by CD4^+^ T cells and ILCs to play a role in intestinal protection.

Gut microbiota metabolites serve as critical mediators linking the microbial community to the host immune system. By modulating specific signaling pathways, these metabolites precisely regulate macrophage polarization, thereby playing a significant role in the transition from colitis to cancer. Notably, the AhR-PI3K/AKT axis and SCFA-mediated HDAC inhibition represent two key molecular mechanisms involved in this process (**Figure 1**).

#### 3.2.1 AhR-PI3K/AKT axis

In the gastrointestinal tract, numerous microbially derived metabolites modulate the activity of the AhR, thereby facilitating metabolic crosstalk between the host and the gut microbiota (Dong and Perdew, 2020). The underlying mechanisms of AhR-microbiota interactions are multifactorial, involving the regulation of immune tolerance and immune responses (Gutiérrez-Vázquez and Quintana, 2018), intestinal homeostasis (Qiu et al., 2012), carcinogenesis (Murray et al., 2014), and maintenance of intestinal barrier integrity (Esser and Rannug, 2015).

The AhR is a ligand-activated transcription factor that resides in the cytoplasm in an inactive, transcriptionally repressed state (Petrulis and Perdew, 2002), Upon ligand binding, the activated AhR translocates to the nucleus and dimerizes with the AhR nuclear translocator (ARNT), forming a functional DNA-binding transcription factor complex (Bersten et al., 2013). AhR plays a crucial role in regulating the adaptive immune response associated with the pathogenesis of IBD (Benson and Shepherd, 2011). Endogenous AhR ligands have been predominantly identified as metabolites derived from Trp and indole metabolism (Dong and Perdew, 2020), such as ILA and indole-3-propionic acid (IPA) (Hubbard et al., 2015). These metabolites modulate the mucosal immune response through activation of the AhR signaling pathway, thereby promoting to the maintenance of intestinal microenvironment (Rothhammer et al., 2016). In DSS-induced colitis mice, IPA binds to AhR to reduce proinflammatory cytokines by mediating IL-10, thereby alleviating disease severity (Rothhammer et al., 2016). ILA may primarily participate in the restoration of intestinal homeostasis by activating the AhR of macrophages (Li et al., 2024). Activation of the PI3K/AKT signaling pathway plays a critical role in modulating excessive macrophage-mediated immune responses and is recognized as a negative regulator of TLR signaling in macrophages (Luyendyk et al., 2008). Using the CAC cell model *in vitro*, it was demonstrated that the addition of ILA can increase AKT phosphorylation without altering the total AKT protein level. By activating the PI3K/AKT signaling pathway, ILA alleviates the inflammatory response and disrupts the differentiation of pro-inflammatory macrophages. In the CAC mouse model, the supplementation of exogenous ILA can increase the proportion of mature colonic macrophages, thereby helping to prevent intestinal tumorigenesis (Li et al., 2024).

In addition, SCFAs promote the production of IL-22 by CD4^+^ T cells and innate lymphoid cells (ILCs) through upregulating AhR and HIF-1α, thereby exerting their protective effects on the intestine (Yang W. et al., 2020). Not only that, ILA exerts anti-inflammatory effects by activating the AhR pathway in intestinal epithelial cells and its downstream Nrf2 pathway. This significantly reduces the production of the pro-inflammatory cytokine IL-8 in intestinal epithelial cell lines and decreases the activation of M1 macrophages, thereby improving gut health (Ehrlich et al., 2020).

#### 3.2.2 SCFAs-HDAC inhibition

Short-chain fatty acids, as microbial metabolites produced by gut microbiota, serve as a primary energy source for intestinal epithelial cells, thereby promoting gastrointestinal health. SCFAs exert immunomodulatory effects by inhibiting the infiltration of inflammatory cells through suppression of HDAC activity (Li et al., 2018). Most HDACs are widely expressed in immune cells, endothelial cells, and vascular smooth muscle cells (Amin et al., 2018).

Among SCFAs, butyrate is the most potent inhibitor of HDACs, followed by propionate (Li et al., 2018). Butyrate and propionate are non-competitive inhibitors of HDACs, exhibiting anti-inflammatory activity by suppressing HDACs activity in macrophages and dendritic cells (Li et al., 2018). HDAC inhibitors have been widely used in cancer therapy, and their anti-inflammatory or immunosuppressive properties have also been reported (Koh et al., 2016). High levels of butyrate present in the intestinal lumen can prevent colorectal cancer and inflammation by inhibiting HDAC, altering the expression of numerous genes with diverse functions, including cell proliferation, apoptosis, and differentiation (Flint et al., 2012). SCFA-mediated HDAC inhibition also serves as an effective anti-inflammatory agent. As a bacterial metabolite abundantly produced in the colon, butyrate suppresses the secretion of inflammatory mediators such as NO and IL-6 by macrophages through inhibition of HDAC activity, thereby contributing to the regulation of intestinal macrophage immune responses and exerting protective effects (Chang et al., 2014). In addition, HDAC inhibitors stimulate anti-inflammatory signaling pathways in endothelial cells, indicating their therapeutic potential in the treatment of inflammatory diseases (Li et al., 2018). Butyrate and propionate also modulate NF-κB activity. Butyrate enhances the production of IL-10 and suppresses the expression of pro-inflammatory molecules such as IL-12, TNF-α, and nitric oxide (NO) by inhibiting NF-κB activation. Propionate reduces NO production in macrophages through suppression of NF-κB activity; however, whether this inhibitory effect is mediated via HDAC inhibition remains to be further elucidated (Usami et al., 2008). Moreover, SCFAs promote the production of IL-22 by CD4^+^ T cells and ILCs through the inhibition of HDACs. The upregulation of IL-22 helps protect the intestines from inflammation caused by intestinal infections and damage (Yang W. et al., 2020). In conclusion, SCFAs play a crucial role in regulating intestinal macrophage function and maintaining intestinal barrier integrity through the HDAC inhibition pathway (Chambers et al., 2018).

## 4 The central role of the microbiota-macrophage interaction network in carcinogenesis

### 4.1 Formation of the early inflammatory microenvironment

**Figure 2:**
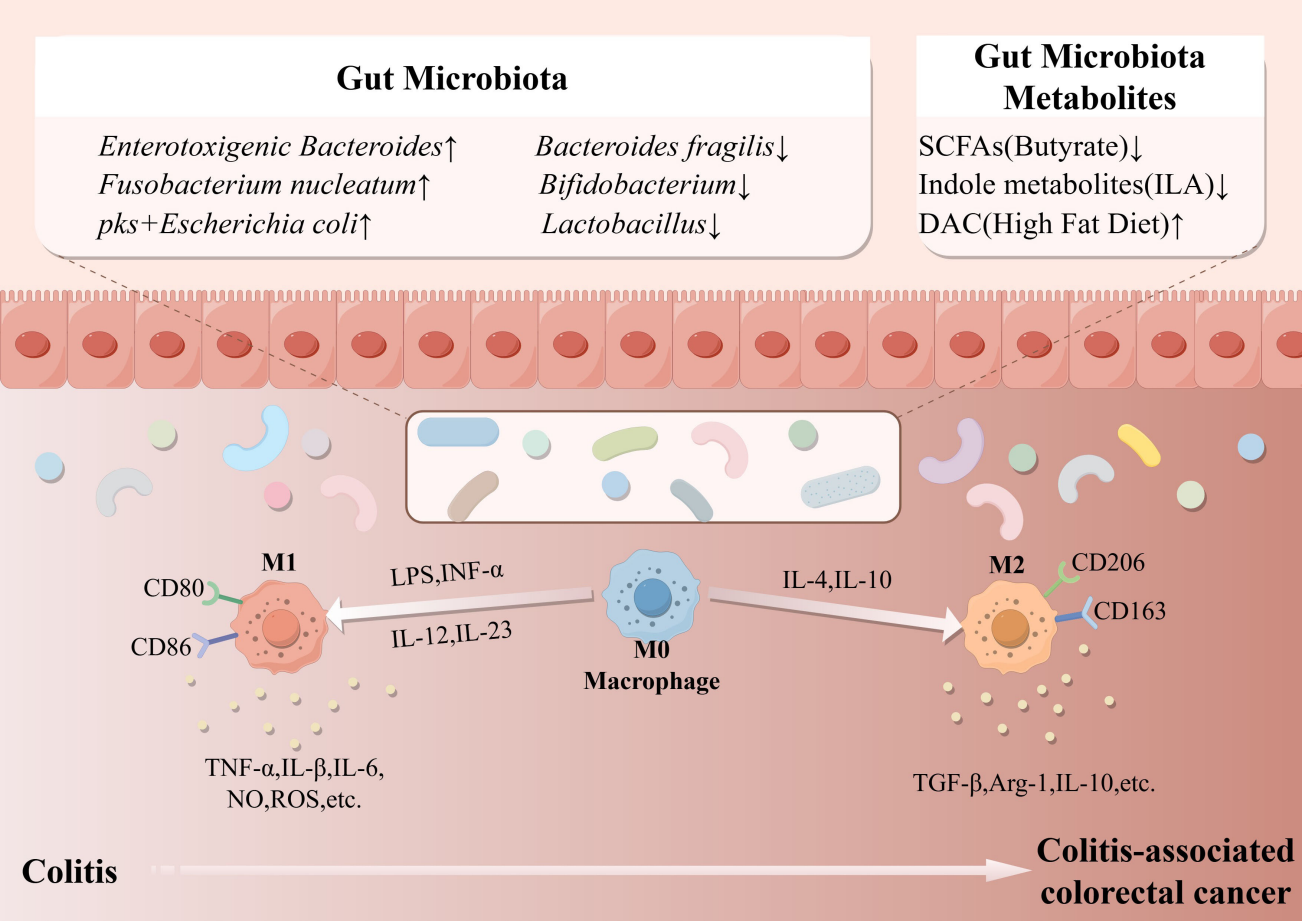
During the process of colitis-associated carcinogenesis, alterations occur in the gut microbiota and their metabolites. The abundance of beneficial commensal bacteria and their metabolites decreases, while that of pathogenic bacteria and pathogenic metabolites increases. Additionally, the polarization of macrophages also changes. During colitis, M1-type macrophages play a major pro-inflammatory role and exacerbate colitis. After carcinogenesis, M2-type macrophages play a tumor-promoting role and exacerbate the carcinogenic process.

The initiation of CAC can be viewed as a self-amplifying inflammatory cycle driven by microbial dysbiosis. Enrichment of pathogenic bacteria such as *F. nucleatum*, *pks^+^* E. coli, and ETBF (Espín et al., 2017), coupled with depletion of beneficial bacteria including *Bifidobacterium*, *Lactobacillus*, and *Bacteroides fragilis* (Hua et al., 2025), fosters a pro-inflammatory intestinal environment. This shift results in elevated levels of pro-inflammatory metabolites such as LPS and DCA, alongside reduced levels of anti-inflammatory metabolites including SCFAs and ILA. In the inflammatory colonic tissue’s immune microenvironment, the frequency of M1-like macrophages, activated dendritic cells (DCs), plasma cell-like DCs, and monocytes are all increased (Liu H. et al., 2019). This dysregulated metabolic environment, acting through receptors such as TLR4 and AhR, not only directly damages the epithelial barrier but also promotes continuous recruitment and polarization of macrophages toward the M1 phenotype. Activated M1 macrophages release substantial amounts of inflammatory mediators, including ROS, IL-6, and TNF-α. By exacerbating tissue damage and promoting DNA mutations, they also activate key pro-inflammatory and pro-carcinogenic signaling pathways, such as NF-κB and STAT3, thereby contributing to the establishment of a microenvironment essential for subsequent dysplasia and carcinogenesis (**Figure 2**).

### 4.2 Interaction between gut microbiota/metabolites and host immunity

The interaction between the host immune system and the gut microbiota is essential for maintaining intestinal homeostasis. Disruption of this crosstalk can directly compromise gut health, contributing to the development of colitis and CAC (Zhao and Jiang, 2021). Compared with healthy individuals, patients with IBD exhibit reduced gut microbial diversity. These alterations in the gut microbiota subsequently lead to changes in microbial metabolites, which may contribute to the pathogenesis of CAC. Clinical studies have shown that changes in the gut microbiota composition of patients with IBD include a significant decrease in the levels of beneficial bacteria such as *Bifidobacterium longum* and butyrate-producing bacteria like *Faecalibacterium prausnitzii* and *Roseburia intestinali*s, while the transcriptional activity of *Clostridium difficile* and the relative abundance of harmful bacteria such as *Enterotoxigenic Bacteroides fragilis* have increased (Vich Vila et al., 2018; Lloyd-Price et al., 2019).

The gut metabolic disorder in patients with IBD is characterized by an imbalance of SCFAs, BAs, and Trp (Qiu et al., 2022). The levels of protective SCFAs, such as butyrate, and Trp metabolites, including indoles, are reduced. This reduction diminishes their capacity to maintain the epithelial barrier and regulate macrophage function through inhibition of HDAC or activation of the AhR, thereby impairing the control of intestinal inflammation (Gonçalves et al., 2018). On the other hand, enriched pro-carcinogenic substances activate specific pro-carcinogenic metabolic signaling pathways. Studies have shown that ETBF suppresses RNA m^6^A modification by inhibiting METTL3 expression, thereby inducing activation of inflammatory macrophages and enhancing the downstream inflammatory response (Yan et al., 2025). Furthermore, ETBF promotes colon tumorigenesis through activation of the Th17-type immune response (Wu et al., 2009; Cao et al., 2021). Under HFD conditions or as a result of gut microbiota dysbiosis, the accumulation of secondary bile acid DCA induces DNA damage in intestinal epithelial cells via oxidative stress and activates the EGFR/Wnt signaling pathway, thereby promoting CRC development (Centuori and Martinez, 2014; Ocvirk and O’Keefe, 2017). Moreover, DCA compromises intestinal barrier integrity, exacerbates microbial imbalance, and enhances the progression of intestinal tumors (Cao et al., 2017).

Notably, the host immune system does not passively accept regulation by the gut microbiota. Rather, it actively modulates the composition and function of the gut microbiota through multiple mechanisms, thereby establishing a dynamic bidirectional feedback loop. Immunoglobulin A (IgA) antibodies and cytokines such as IL-22, secreted by intestinal epithelial cells and immune cells in the lamina propria, can selectively inhibit or promote the colonization and proliferation of specific bacterial species.

IgA is the predominant antibody subtype involved in mucosal immunity and primarily exists in the form of secretory polymeric IgA (sIgA), serving as a key mediator of intestinal immune responses (Corthésy, 2013; Herr, 2020). sIgA can specifically recognize certain members of the gut microbiota, facilitating their attachment to the intestinal mucosa and promoting their colonization by excluding exogenous microbial competitors (Donaldson et al., 2018). For instance, BAs have been shown to enhance the stable colonization and mucosal adhesion of ETBF through modulation of sIgA levels (Guo et al., 2022). Studies have demonstrated that IgA deficiency accelerates the onset and progression of CAC. The intestinal microbiota composition, shaped by IgA levels, ultimately determines the severity of intestinal inflammation and the advancement of CAC (Tang et al., 2024).

The interaction between the microbiota and IL-22 plays a central role in the homeostasis regulation at the intestinal barrier site (Yang W. et al., 2020). IL-22 strengthens the intestinal mucosal barrier and promotes epithelial repair by activating the STAT3 signaling pathway in epithelial cells, leading to the production of antimicrobial peptides such as RegIIIγ, mucins, and chemokines (Kirchberger et al., 2013). RegIIIγ selectively binds to and eliminates specific Gram-positive bacteria in close proximity to the mucosal surface, including *Listeria innocua* and *Enterococcus faecalis*, thereby contributing to host defense against bacterial pathogens (Cash et al., 2006). The IL-22 signaling pathway is likely to play a critical role in controlling bacterial infections in the human gastrointestinal tract, particularly those caused by attaching and effacing pathogens (Zheng et al., 2008). Beyond immune effector molecules, the inflammatory microenvironment itself can reshape the gut microbial ecosystem. In the context of chronic inflammation observed in IBD, elevated levels of ROS and nitrates generate a pro-oxidative environment that is detrimental to the survival of most obligate anaerobic beneficial bacteria, while favoring the expansion of potentially pathogenic facultative anaerobes such as *Enterobacteriaceae*, thereby exacerbating microbial dysbiosis (Winter et al., 2013).

In conclusion, the interaction between the gut microbiota and the host immune system is central to maintaining intestinal homeostasis. Dysregulation of this interaction not only disrupts microbial community structure and metabolic function but also promotes the initiation and progression of intestinal inflammation and tumorigenesis through the synergistic effects of multiple pathways. Preserving the balance of this crosstalk may provide critical therapeutic targets for the prevention and treatment of associated gastrointestinal disorders.

## 5 Intervention strategies targeting interaction networks

Based on the understanding of the central role played by the microbiota-macrophage interaction network in CAC, therapeutic intervention in this network has become a highly promising strategy. Key approaches include modulation of the gut microbiota, application of advanced delivery technologies, and direct reprogramming of immune cells (**Table 2**).

**Table 2:** Summary of CAC-related treatment strategies.

Treatment strategy	Mechanism of action	Representative methods	Evidence type	Limitations
5.1 Probiotic and Metabolite Supplementation	Regulate the composition of gut microbiota and increase the abundance of beneficial bacteria.	Oral administration of probiotics, supplementation of dietary fiber, or intake of short-chain fatty acids.	Mouse model/clinical	The therapeutic effects vary among individuals, and the optimal treatment plan requires further confirmation.
5.2 Fecal microbiota transplantation	Restore the diversity and balance of gut microbiota; repair epithelial function.	Single or multiple FMT infusions, and multi-donor FMT combined with an anti-inflammatory diet.	Mouse model/clinical	lack of large-scale randomized controlled trial (RCT) evidence for patients with CAC, and the standardized treatment protocol needs to be improved.
5.3 Nanomedicine for Drug Delivery	Achieve precise drug delivery to the colonic region; increase local drug concentration and reduce systemic toxicity.	pH-responsive hydrogels, anti-EGFR antibody-modified nanoparticles, CUR-assembled nanoparticles.	IBD/CAC/CRC mouse model	Regulatory barriers and difficulties in achieving manufacturing scale-up pose challenges, and further research and development are still required for clinical applications.
5.4 Macrophage-Targeted Therapy	Regulate M1/M2 polarization of macrophages and inhibit inflammatory pathways.	Dihydroartemisinin, cetuximab, dioscin, etc.	IBD/CAC/CRC mouse model	Most of them are in the pre-clinical research stage, and their clinical potential remains to be verified.

### 5.1 Probiotics and metabolite supplementation

Alterations in the gut microbiota are closely associated with tumorigenesis. The use of probiotics to maintain a low abundance of potentially pathogenic microbial species may represent an effective strategy for mitigating CAC (Chung et al., 2021; Li et al., 2024). Oral administration of antibiotics, bacterial metabolites, and probiotic supplements has been shown to modulate the composition and function of the gut microbiota (Gordon et al., 2022; Wang et al., 2023). For example, oral supplementation with *B. breve lw01* has been demonstrated to delay the onset of CAC by attenuating the colonic inflammatory response, reducing macrophage infiltration, and promoting the differentiation of immature colonic macrophages. Dietary fiber or SCFAs supplementation has exhibited therapeutic benefits in the management of colitis in both clinical and experimental settings (Lee et al., 2022). Therefore, microbiome-targeted interventions hold significant promise for restoring immune homeostasis and suppressing the development of CAC.

### 5.2 Fecal microbiota transplantation

Fecal microbiota transplantation (FMT) transfers gut microbiota from healthy donors to recipients to restore microbial balance (Luo et al., 2025). It provides protection against bacterial translocation and slows disease progression by introducing a diverse microbial community and restoring the epithelial defense system (Wardill et al., 2019). Unlike antibiotic and probiotic therapies, FMT increases the diversity of the recipient’s fecal bacterial population. For patients with IBD, it may represent a more effective therapeutic approach for modulating the gut microbiota. Multi-donor FMT combined with an anti-inflammatory diet has effectively induced deep remission in patients with mild to moderate ulcerative colitis (Kedia et al., 2022). Furthermore, the FMT approach to modulating gut microbiota has successfully cured patients with refractory immune checkpoint inhibitor-associated colitis (Wang et al., 2018). Analysis of gut microbiota and metabolomics indicates that FMT can reverse the imbalance of dominant gut microbiota and metabolic disorders in CRC mouse models, thereby alleviating the severity of CRC. Further research on the CAC mouse model reveals that gut microbiota plays a crucial regulatory role in the occurrence of CAC, and FMT treatment can either mitigate or exacerbate colitis-induced carcinogenesis (Song et al., 2024). As a promising therapeutic approach for colorectal cancer, FMT modulates the composition and structure of the gut microbiota, reduces excessive colonic macrophage infiltration, and improves disease progression and clinical symptoms (Hua et al., 2025). However, larger randomized controlled trials are still needed to better define the role of FMT in the treatment of CAC.

### 5.3 Nanomedicine for drug delivery

Clinical pathological studies have indicated that the intestinal mucosal immune response and intestinal microbiota dysbiosis are closely associated with the occurrence and development of intestinal diseases (Citi, 2018). Current therapeutic strategies for IBD primarily focus on suppressing hyperactive immune responses, scavenging intestinal ROS, and modulating the gut microbiota. However, due to the complex anatomy of the gastrointestinal tract and the presence of the mucus barrier, small-molecule and biologic agents often fail to reach the inflamed colonic sites effectively, resulting in limited therapeutic efficacy and adverse effects (Zhang B. et al., 2023; Fu et al., 2024). Therefore, the development of advanced therapeutic approaches capable of simultaneously regulating the gut microbiota and attenuating excessive immune activation is essential. The integration of nanotechnology with microbiota-targeted interventions offers a promising strategy for achieving this goal. Lei et al. designed a pH-responsive hydrogel that selectively releases anti-inflammatory drugs in the colonic region of a mouse model of UC. By reducing the expression of TNF-α and IL-6, increasing the expression of IL-10, scavenging ROS in macrophages, and improving the expression of intestinal mucosal tight junction proteins, this hydrogel effectively improves the colonic inflammatory microenvironment (Lei et al., 2023). Djermane et al. utilized EGFR antibody-functionalized nanoparticles to deliver cholinesterase-based therapeutics to colorectal cancer cells, thereby enhancing tumor-specific accumulation and reducing off-target toxicity (Djermane et al., 2024). Han et al. assembled water-insoluble curcumin and 7-ethyl-10-hydroxycamptothecin into stable nanoparticles. Oral administration of these nanoparticles enabled accumulation in inflamed intestinal regions and tumor tissues. By reducing inflammatory cytokines and ROS levels in macrophages, they protected mice from UC and CAC (Han et al., 2019). In summary, nanotechnology holds significant potential for enabling the development of more precise, controllable, and site-specific drug delivery systems for treating complex gastrointestinal disorders such as IBD and CAC.

### 5.4 Macrophage-targeted therapy

Macrophages are among the most abundant immune cell populations in the colonic tumor microenvironment and play a critical role in tumor initiation, promotion, and invasion (Steinbach and Plevy, 2014; Yang Y. et al., 2020). Therefore, modulating macrophage polarization to mitigate their tumor-promoting functions represents a promising therapeutic strategy for improving outcomes in CAC. Prior to tumor development, M1 macrophages exert pro-inflammatory effects that contribute to tumorigenesis. Dihydroartemisinin suppresses M1 macrophage polarization through inhibition of the TLR4 signaling pathway, thereby attenuating intestinal inflammation (Bai et al., 2021). After the formation of CAC, M1 polarization inhibits the progression of CAC through tumor immunity, while M2 polarization promotes tumor progression and metastasis. Therefore, reducing the proportion of M2 polarization can be considered as a viable therapeutic approach (Grimm et al., 2011). Cetuximab, an EGFR-targeting monoclonal antibody, can counteract the pro-tumorigenic activities of macrophages in CRC (Zhang et al., 2016). Dioscin inhibits M2 macrophage polarization, leading to suppression of tumorigenesis in CAC (Xun et al., 2023). YTE17, an active fraction derived from Garcinia yunnanensis, suppresses M2 polarization by downregulating the JNK, STAT3, and ERK signaling pathways, thereby inhibiting tumor development in CAC (Sui et al., 2020). Triptolide inhibits the polarization of M2 macrophages and reduces the secretion of anti-inflammatory cytokines, exerting an anti-tumor effect by suppressing the tumor-promoting effect of M2 macrophages on CAC (Li et al., 2020b; Li et al., 2020a). However, further preclinical and clinical studies are required to evaluate the translational potential of these agents.

## 6 Conclusions and future prospects

CAC as a typical inflammation-driven cancer, resulting from a vicious cycle of persistent inflammatory response and immunosuppression. Key factors such as inflammatory mediators and microbial metabolites exert regulatory effects at various stages of disease progression through their pro-inflammatory or anti-inflammatory properties. In this context, gut microbiota imbalance and the disturbance of their metabolites represent central mechanisms driving the inflammation-to-cancer transition, offering a promising target for preventing and treating CAC by modulating the microbe-immune system axis. However, translating these insights into clinical practice remains challenging, and these challenges highlight key directions for future research.

1. Unclear core mechanism: The gut microbiota and metabolites play a crucial role in the development of CAC. However, the mechanism by which they perform synergistic, antagonistic, or cascade regulation remains unclear. In the future, it is necessary to systematically analyze the microbiota-metabolite association networks at different stages of CAC occurrence by means of multi-omics integrated analysis and bioinformatics methods.
2. Limitations of current models: Human CAC cases are relatively rare, and obtaining clinical samples is challenging. Existing animal models (such as DSS/AOM) inevitably fall short in simulating the pathological progression of CAC compared to its human counterpart. Future efforts should focus on developing translational models that more closely mimic human CAC pathology. This could involve co-culturing patient-derived organoids, immune cells, and microbial communities to establish experimental systems capable of reproducing the complexity of the human microenvironment.
3. Difficulties in targeted delivery: How to achieve the targeted delivery of probiotics, metabolites, or targeted drugs to enhance their bioavailability and stability at intestinal lesion sites remains a significant challenge in translational research. In the future, efforts should be dedicated to developing intelligent nanodelivery systems that can simultaneously respond to the inflammatory microenvironment and target tumor-associated macrophages, so as to achieve the precise delivery of drugs or microbiota to the diseased sites and improve the precision and effectiveness of treatment.

Despite the significant challenges, with the advancement of the aforementioned research directions, we are expected to precisely analyze the gut microbiota and their metabolic effector molecules involved in the pathogenesis of CAC in the future. This progress not only deepens our understanding of the underlying pathophysiological mechanisms but also lays a solid theoretical foundation for personalized prevention and treatment strategies based on microbial metabolism.

## References

[B1] Abrantes F. A. Nascimento B. B. Andrade M. E. R. de Barros P. A. V. Cartelle C. T. Martins F. S. . (2020). Treatment with Bifidobacterium longum 5(1A) attenuates intestinal damage and inflammatory response in experimental colitis. Benef. Microbes 11, 1. doi: 10.3920/bm2019.0098, PMID: 32066260

[B2] Amin S. A. Adhikari N. Jha T. (2018). Structure-activity relationships of HDAC8 inhibitors: Non-hydroxamates as anticancer agents. Pharmacol. Res. 131, 5. doi: 10.1016/j.phrs.2018.03.001, PMID: 29514055

[B3] Aminin D. Wang Y. M. (2021). Macrophages as a “weapon” in anticancer cellular immunotherapy. Kaohsiung. J. Med. Sci. 37, 9. doi: 10.1002/kjm2.12405, PMID: 34110692 PMC11896134

[B4] Arboleya S. Watkins C. Stanton C. Ross R. P. (2016). Gut bifidobacteria populations in human health and aging. Front. Microbiol. 7. doi: 10.3389/fmicb.2016.01204, PMID: 27594848 PMC4990546

[B5] Arthur J. C. Perez-Chanona E. Mühlbauer M. Tomkovich S. Uronis J. M. Fan T. J. . (2012). Intestinal inflammation targets cancer-inducing activity of the microbiota. Science 338, 6103. doi: 10.1126/science.1224820, PMID: 22903521 PMC3645302

[B6] Azizi E. Carr A. J. Plitas G. Cornish A. E. Konopacki C. Prabhakaran S. . (2018). Single-cell map of diverse immune phenotypes in the breast tumor microenvironment. Cell 174, 5. doi: 10.1016/j.cell.2018.05.060, PMID: 29961579 PMC6348010

[B7] Baars J. E. Looman C. W. Steyerberg E. W. Beukers R. Tan A. C. Weusten B. L. . (2011). The risk of inflammatory bowel disease-related colorectal carcinoma is limited: results from a nationwide nested case-control study. Am. J. Gastroenterol. 106, 2. doi: 10.1038/ajg.2010.428, PMID: 21045815

[B8] Bai B. Wu F. Ying K. Xu Y. Shan L. Lv Y. . (2021). Therapeutic effects of dihydroartemisinin in multiple stages of colitis-associated colorectal cancer. Theranostics 11, 13. doi: 10.7150/thno.55939, PMID: 33995655 PMC8120200

[B9] Bao X. Wang D. Dai X. Liu C. Zhang H. Jin Y. . (2023). An immunometabolism subtyping system identifies S100A9(+) macrophage as an immune therapeutic target in colorectal cancer based on multiomics analysis. Cell Rep. Med. 4, 4. doi: 10.1016/j.xcrm.2023.100987, PMID: 36990096 PMC10140461

[B10] Benson J. M. Shepherd D. M. (2011). Aryl hydrocarbon receptor activation by TCDD reduces inflammation associated with Crohn’s disease. Toxicol. Sci. 120, 1. doi: 10.1093/toxsci/kfq360, PMID: 21131560 PMC3044199

[B11] Bersten D. C. Sullivan A. E. Peet D. J. Whitelaw M. L. (2013). bHLH-PAS proteins in cancer. Nat. Rev. Cancer. 13, 12. doi: 10.1038/nrc3621, PMID: 24263188

[B12] Bode J. G. Ehlting C. Haussinger D. (2012). The macrophage response towards LPS and its control through the p38(MAPK)-STAT3 axis. Cell Signal. 24, 6. doi: 10.1016/j.cellsig.2012.01.018, PMID: 22330073

[B13] Boleij A. Hechenbleikner E. M. Goodwin A. C. Badani R. Stein E. M. Lazarev M. G. . (2015). The Bacteroides fragilis toxin gene is prevalent in the colon mucosa of colorectal cancer patients. Clin. Infect. Dis. 60, 2. doi: 10.1093/cid/ciu787, PMID: 25305284 PMC4351371

[B14] Bottacini F. van Sinderen D. Ventura M. (2017). Omics of bifidobacteria: research and insights into their health-promoting activities. Biochem. J. 474, 24. doi: 10.1042/bcj20160756, PMID: 29212851

[B15] Boutilier A. J. Elsawa S. F. (2021). Macrophage polarization states in the tumor microenvironment. Int. J. Mol. Sci. 22, 13. doi: 10.3390/ijms22136995, PMID: 34209703 PMC8268869

[B16] Cao H. Luo S. Xu M. Zhang Y. Song S. Wang S. . (2014). The secondary bile acid, deoxycholate accelerates intestinal adenoma-adenocarcinoma sequence in Apc (min/+) mice through enhancing Wnt signaling. Fam. Cancer. 13, 4. doi: 10.1007/s10689-014-9742-3, PMID: 25106466

[B17] Cao H. Xu M. Dong W. Deng B. Wang S. Zhang Y. . (2017). Secondary bile acid-induced dysbiosis promotes intestinal carcinogenesis. Int. J. Cancer. 140, 11. doi: 10.1002/ijc.30643, PMID: 28187526

[B18] Cao Y. Wang Z. Yan Y. Ji L. He J. Xuan B. . (2021). Enterotoxigenic bacteroidesfragilis promotes intestinal inflammation and Malignancy by inhibiting exosome-packaged miR-149-3p. Gastroenterology 161, 5. doi: 10.1053/j.gastro.2021.08.003, PMID: 34371001

[B19] Cash H. L. Whitham C. V. Behrendt C. L. Hooper L. V. (2006). Symbiotic bacteria direct expression of an intestinal bactericidal lectin. Science 313, 5790. doi: 10.1126/science.1127119, PMID: 16931762 PMC2716667

[B20] Centuori S. M. Martinez J. D. (2014). Differential regulation of EGFR-MAPK signaling by deoxycholic acid (DCA) and ursodeoxycholic acid (UDCA) in colon cancer. Dig. Dis. Sci. 59, 10. doi: 10.1007/s10620-014-3190-7, PMID: 25027205 PMC4163523

[B21] Chai N. Xiong Y. Zhang Y. Cheng Y. Shi W. Yao Y. . (2021). YYFZBJS inhibits colorectal tumorigenesis by remodeling gut microbiota and influence on M2 macrophage polarization *in vivo* and *in vitro*. Am. J. Cancer Res. 11, 11. doi: 10.2139/ssrn.3837225, PMID: 34873464 PMC8640793

[B22] Chambers E. S. Preston T. Frost G. Morrison D. J. (2018). Role of gut microbiota-generated short-chain fatty acids in metabolic and cardiovascular health. Curr. Nutr. Rep. 7, 4. doi: 10.1007/s13668-018-0248-8, PMID: 30264354 PMC6244749

[B23] Chandrasekaran P. Weiskirchen S. Weiskirchen R. (2024). Effects of probiotics on gut microbiota: an overview. Int. J. Mol. Sci. 25, 11. doi: 10.3390/ijms25116022, PMID: 38892208 PMC11172883

[B24] Chang P. V. Hao L. Offermanns S. Medzhitov R. (2014). The microbial metabolite butyrate regulates intestinal macrophage function via histone deacetylase inhibition. Proc. Natl. Acad. Sci. U. S. A. 111, 6. doi: 10.1073/pnas.1322269111, PMID: 24390544 PMC3926023

[B25] Chen Y. Wang B. Yuan X. Lu Y. Hu J. Gao J. . (2021). Vitexin prevents colitis-associated carcinogenesis in mice through regulating macrophage polarization. Phytomedicine 83, 4. doi: 10.1016/j.phymed.2021.153489, PMID: 33571919

[B26] Chiu Y. H. Hsieh Y. J. Liao K. W. Peng K. C. (2010). Preferential promotion of apoptosis of monocytes by Lactobacillus casei rhamnosus soluble factors. Clin. Nutr. 29, 1. doi: 10.1016/j.clnu.2009.07.004, PMID: 19665262

[B27] Choi M. Lee Y. Lee N. K. Bae C. H. Park D. C. Paik H. D. . (2019). Immunomodulatory effects by bifidobacterium longum KACC 91563 in mouse splenocytes and macrophages. J. Microbiol. Biotechnol. 29, 11. doi: 10.4014/jmb.1812.12002, PMID: 31546293

[B28] Chung Y. Ryu Y. An B. C. Yoon Y. S. Choi O. Kim T. Y. . (2021). A synthetic probiotic engineered for colorectal cancer therapy modulates gut microbiota. Microbiome 9, 1. doi: 10.1186/s40168-021-01071-4, PMID: 34039418 PMC8157686

[B29] Citi S. (2018). Intestinal barriers protect against disease. Science 359, 6380. doi: 10.1126/science.aat0835, PMID: 29590026

[B30] Coburn L. A. Singh K. Asim M. Barry D. P. Allaman M. M. Al-Greene N. T. . (2019). Loss of solute carrier family 7 member 2 exacerbates inflammation-associated colon tumorigenesis. Oncogene 38, 7. doi: 10.1038/s41388-018-0492-9, PMID: 30202097 PMC6377304

[B31] Corthésy B. (2013). Multi-faceted functions of secretory IgA at mucosal surfaces. Front. Immunol. 4. doi: 10.3389/fimmu.2013.00185, PMID: 23874333 PMC3709412

[B32] Cuevas-Ramos G. Petit C. R. Marcq I. Boury M. Oswald E. Nougayrède J. P. (2010). Escherichia coli induces DNA damage *in vivo* and triggers genomic instability in mammalian cells. Proc. Natl. Acad. Sci. U. S. A. 107, 25. doi: 10.1073/pnas.1001261107, PMID: 20534522 PMC2895108

[B33] Cui Q. Y. Tian X. Y. Liang X. Zhang Z. Wang R. Zhou Y. . (2022). Bifidobacterium bifidum relieved DSS-induced colitis in mice potentially by activating the aryl hydrocarbon receptor. Food Funct. 13, 9. doi: 10.1039/d1fo04219j, PMID: 35416187

[B34] Dai L. Liu Y. Yin Y. Li J. Dong Z. Chen N. . (2020). SARI suppresses colitis-associated cancer development by maintaining MCP-1-mediated tumour-associated macrophage recruitment. J. Cell Mol. Med. 24, 1. doi: 10.1111/jcmm.14699, PMID: 31578820 PMC6933368

[B35] DeNardo D. G. Ruffell B. (2019). Macrophages as regulators of tumour immunity and immunotherapy. Nat. Rev. Immunol. 19, 6. doi: 10.1038/s41577-019-0127-6, PMID: 30718830 PMC7339861

[B36] Deng Y. Jia X. Liu L. He Q. Liu L. (2025). The role of intestinal macrophage polarization in colitis-associated colon cancer. Front. Immunol. 16. doi: 10.3389/fimmu.2025.1537631, PMID: 40109347 PMC11919874

[B37] Dias A. M. M. Douhard R. Hermetet F. Regimbeau M. Lopez T. E. Gonzalez D. . (2021). Lactobacillus stress protein GroEL prevents colonic inflammation. J. Gastroenterol. 56, 5. doi: 10.1007/s00535-021-01774-3, PMID: 33782752

[B38] Dixit K. Chaudhari D. Dhotre D. Shouche Y. Saroj S. (2021). Restoration of dysbiotic human gut microbiome for homeostasis. Life Sci. 278,15. doi: 10.1016/j.lfs.2021.119622, PMID: 34015282

[B39] Djermane R. Nieto C. Vega M. A. Del Valle E. M. M. (2024). EGFR-targeting polydopamine nanoparticles co-loaded with 5-fluorouracil, irinotecan, and leucovorin to potentially enhance metastatic colorectal cancer therapy. Sci. Rep. 14, 1. doi: 10.1038/s41598-024-80879-0, PMID: 39587206 PMC11589782

[B40] Domínguez-Acosta O. Vega L. Estrada-Muñiz E. Rodríguez M. S. Gonzalez F. J. Elizondo G. (2018). Activation of aryl hydrocarbon receptor regulates the LPS/IFNγ-induced inflammatory response by inducing ubiquitin-proteosomal and lysosomal degradation of RelA/p65. Biochem. Pharmacol. 155, 9. doi: 10.1016/j.bcp.2018.06.016, PMID: 29935959 PMC6594173

[B41] Donaldson G. P. Ladinsky M. S. Yu K. B. Sanders J. G. Yoo B. B. Chou W. C. . (2018). Gut microbiota utilize immunoglobulin A for mucosal colonization. Science 360, 6390. doi: 10.1126/science.aaq0926, PMID: 29724905 PMC5973787

[B42] Dong F. Perdew G. H. (2020). The aryl hydrocarbon receptor as a mediator of host-microbiota interplay. Gut. Microbes 12, 1. doi: 10.1080/19490976.2020.1859812, PMID: 33382356 PMC7781536

[B43] Dong Y. Yang Q. Niu R. Zhang Z. Huang Y. Bi Y. . (2022). Modulation of tumor-associated macrophages in colitis-associated colorectal cancer. J. Cell Physiol. 237, 12. doi: 10.1002/jcp.30906, PMID: 36302153

[B44] Ehrlich A. M. Pacheco A. R. Henrick B. M. Taft D. Xu G. Huda M. N. . (2020). Indole-3-lactic acid associated with Bifidobacterium-dominated microbiota significantly decreases inflammation in intestinal epithelial cells. BMC Microbiol. 20, 1. doi: 10.1186/s12866-020-02023-y, PMID: 33225894 PMC7681996

[B45] Eom Y. W. Akter R. Li W. Lee S. Hwang S. Kim J. . (2020). M1 macrophages promote TRAIL expression in adipose tissue-derived stem cells, which suppresses colitis-associated colon cancer by increasing apoptosis of CD133(+) cancer stem cells and decreasing M2 macrophage population. Int. J. Mol. Sci. 21, 11. doi: 10.3390/ijms21113887, PMID: 32485960 PMC7312348

[B46] Espín J. C. González-Sarrías A. Tomás-Barberán F. A. (2017). The gut microbiota: A key factor in the therapeutic effects of (poly)phenols. Biochem. Pharmacol. 139, 17. doi: 10.1016/j.bcp.2017.04.033, PMID: 28483461

[B47] Esser C. Rannug A. (2015). The aryl hydrocarbon receptor in barrier organ physiology, immunology, and toxicology. Pharmacol. Rev. 67, 2. doi: 10.1124/pr.114.009001, PMID: 25657351

[B48] Fang Z. Pan T. Li L. Wang H. Zhu J. Zhang H. . (2022). Bifidobacterium longum mediated tryptophan metabolism to improve atopic dermatitis via the gut-skin axis. Gut. Microbes 14, 1. doi: 10.1080/19490976.2022.2044723, PMID: 35239463 PMC8903757

[B49] Feng X. Wang K. Cao S. Ding L. Qiu F. (2020). Pharmacokinetics and excretion of berberine and its nine metabolites in rats. Front. Pharmacol. 11. doi: 10.3389/fphar.2020.594852, PMID: 33584274 PMC7874128

[B50] Flint H. J. Scott K. P. Louis P. Duncan S. H. (2012). The role of the gut microbiota in nutrition and health. Nat. Rev. Gastroenterol. Hepatol. 9, 10. doi: 10.1038/nrgastro.2012.156, PMID: 22945443

[B51] Fu Y. J. Zhao X. Wang L. Y. Li K. Jiang N. Zhang S. T. . (2024). A gas therapy strategy for intestinal flora regulation and colitis treatment by nanogel-based multistage NO delivery microcapsules. Adv. Mater. 36, 19. doi: 10.1002/adma.202309972, PMID: 38324725

[B52] Gandhi C. R. (2020). Pro- and anti-fibrogenic functions of gram-negative bacterial lipopolysaccharide in the liver. Front. Med. (Lausanne). 7. doi: 10.3389/fmed.2020.00130, PMID: 32373617 PMC7186417

[B53] Garrido D. Ruiz-Moyano S. Kirmiz N. Davis J. C. Totten S. M. Lemay D. G. . (2016). A novel gene cluster allows preferential utilization of fucosylated milk oligosaccharides in Bifidobacterium longum subsp. longum SC596. Sci. Rep. 6, 6. doi: 10.1038/srep35045, PMID: 27756904 PMC5069460

[B54] Gonçalves P. Araújo J. R. Di Santo J. P. (2018). A cross-talk between microbiota-derived short-chain fatty acids and the host mucosal immune system regulates intestinal homeostasis and inflammatory bowel disease. Inflammation Bowel. Dis. 24, 3. doi: 10.1093/ibd/izx029, PMID: 29462379

[B55] Gordon M. Sinopoulou V. Grafton-Clarke C. Akobeng A. K. (2022). Antibiotics for the induction and maintenance of remission in ulcerative colitis. Cochrane Database Syst. Rev. 5, 5. doi: 10.1002/14651858.CD013743.pub2, PMID: 35583095 PMC9115763

[B56] Grimm M. Lazariotou M. Kircher S. Höfelmayr A. Germer C. T. von Rahden B. H. . (2011). Tumor necrosis factor-α is associated with positive lymph node status in patients with recurrence of colorectal cancer-indications for anti-TNF-α agents in cancer treatment. Cell Oncol. (Dordr). 34, 4. doi: 10.1007/s13402-011-0027-7, PMID: 21573932 PMC12994998

[B57] Grivennikov S. Karin E. Terzic J. Mucida D. Yu G. Y. Vallabhapurapu S. . (2009). IL-6 and Stat3 are required for survival of intestinal epithelial cells and development of colitis-associated cancer. Cancer Cell. 15, 2. doi: 10.1016/j.ccr.2009.01.001, PMID: 19185845 PMC2667107

[B58] Guo S. Peng Y. Lou Y. Cao L. Liu J. Lin N. . (2022). Downregulation of the farnesoid X receptor promotes colorectal tumorigenesis by facilitating enterotoxigenic Bacteroides fragilis colonization. Pharmacol. Res. 177, 3. doi: 10.1016/j.phrs.2022.106101, PMID: 35104632

[B59] Guo X. Shin V. Y. Cho C. H. (2001). Modulation of heme oxygenase in tissue injury and its implication in protection against gastrointestinal diseases. Life Sci. 69, 25–26. doi: 10.1016/s0024-3205(01)01417-5, PMID: 11758836

[B60] Guo Z. Wen Z. Qin A. Zhou Y. Liao Z. Liu Z. . (2013). Antisense oligonucleotide treatment enhances the recovery of acute lung injury through IL-10-secreting M2-like macrophage-induced expansion of CD4+ regulatory T cells. J. Immunol. 190, 8. doi: 10.4049/jimmunol.1203233, PMID: 23514739 PMC3619531

[B61] Gutiérrez-Vázquez C. Quintana F. J. (2018). Regulation of the immune response by the aryl hydrocarbon receptor. Immunity 48, 1. doi: 10.1016/j.immuni.2017.12.012, PMID: 29343438 PMC5777317

[B62] Hamer H. M. Jonkers D. Venema K. Vanhoutvin S. Troost F. J. Brummer R. J. (2008). Review article: the role of butyrate on colonic function. Aliment. Pharmacol. Ther. 27, 2. doi: 10.1111/j.1365-2036.2007.03562.x, PMID: 17973645

[B63] Han W. Xie B. Li Y. Shi L. Wan J. Chen X. . (2019). Orally deliverable nanotherapeutics for the synergistic treatment of colitis-associated colorectal cancer. Theranostics 9, 24. doi: 10.7150/thno.38081, PMID: 31695780 PMC6831307

[B64] Hardbower D. M. Coburn L. A. Asim M. Singh K. Sierra J. C. Barry D. P. . (2017). EGFR-mediated macrophage activation promotes colitis-associated tumorigenesis. Oncogene 36, 27. doi: 10.1038/onc.2017.23, PMID: 28263971 PMC5501754

[B65] Herr A. B. (2020). Secret(ory) revealed: the long-awaited structures of secretory IgA. Cell Res. 30, 7. doi: 10.1038/s41422-020-0351-4, PMID: 32523108 PMC7343791

[B66] Hill C. Guarner F. Reid G. Gibson G. R. Merenstein D. J. Pot B. . (2014). Expert consensus document. The International Scientific Association for Probiotics and Prebiotics consensus statement on the scope and appropriate use of the term probiotic. Nat. Rev. Gastroenterol. Hepatol. 11, 8. doi: 10.1038/nrgastro.2014.66, PMID: 24912386

[B67] Hou H. Chen D. Zhang K. Zhang W. Liu T. Wang S. . (2022). Gut microbiota-derived short-chain fatty acids and colorectal cancer: Ready for clinical translation? Cancer Lett. 526, 3. doi: 10.1016/j.canlet.2021.11.027, PMID: 34843863

[B68] Hu Y. Chen Z. Xu C. Kan S. Chen D. (2022). Disturbances of the gut microbiota and microbiota-derived metabolites in inflammatory bowel disease. Nutrients 14, 23. doi: 10.3390/nu14235140, PMID: 36501169 PMC9735443

[B69] Hua D. Yang Q. Li X. Zhou X. Kang Y. Zhao Y. . (2025). The combination of Clostridium butyricum and Akkermansia muciniphila mitigates DSS-induced colitis and attenuates colitis-associated tumorigenesis by modulating gut microbiota and reducing CD8(+) T cells in mice. mSystems 10, 2. doi: 10.1128/msystems.01567-24, PMID: 39840995 PMC11834468

[B70] Huang W. Cho K. Y. Meng D. Walker W. A. (2021). The impact of indole-3-lactic acid on immature intestinal innate immunity and development: a transcriptomic analysis. Sci. Rep. 11, 1. doi: 10.1038/s41598-021-87353-1, PMID: 33850185 PMC8044159

[B71] Huang C. Tan H. Song M. Liu K. Liu H. Wang J. . (2023). Maternal Western diet mediates susceptibility of offspring to Crohn’s-like colitis by deoxycholate generation. Microbiome 11, 1. doi: 10.1186/s40168-023-01546-6, PMID: 37131223 PMC10155335

[B72] Hubbard T. D. Murray I. A. Perdew G. H. (2015). Indole and tryptophan metabolism: endogenous and dietary routes to ah receptor activation. Drug Metab. Dispos. 43, 10. doi: 10.1124/dmd.115.064246, PMID: 26041783 PMC4576673

[B73] Hwang S. Yi H. C. Hwang S. Jo M. Rhee K. J. (2020). Dietary Salt Administration Decreases Enterotoxigenic Bacteroides fragilis (ETBF)-Promoted Tumorigenesis via Inhibition of Colonic Inflammation. Int. J. Mol. Sci. 21, 21. doi: 10.3390/ijms21218034, PMID: 33126615 PMC7663446

[B74] Iftekhar A. Berger H. Bouznad N. Heuberger J. Boccellato F. Dobrindt U. . (2021). Genomic aberrations after short-term exposure to colibactin-producing E. coli transform primary colon epithelial cells. Nat. Commun. 12, 1. doi: 10.1038/s41467-021-21162-y, PMID: 33579932 PMC7881031

[B75] Italiani P. Boraschi D. (2014). From monocytes to M1/M2 macrophages: phenotypical vs. Functional differentiation. Front. Immunol. 5. doi: 10.3389/fimmu.2014.00514, PMID: 25368618 PMC4201108

[B76] Jin X. You L. Qiao J. Han W. Pan H. (2024). Autophagy in colitis-associated colon cancer: exploring its potential role in reducing initiation and preventing IBD-Related CAC development. Autophagy 20, 2. doi: 10.1080/15548627.2023.2259214, PMID: 37723664 PMC10813649

[B77] Joyce S. A. Gahan C. G. (2016). Bile acid modifications at the microbe-host interface: potential for nutraceutical and pharmaceutical interventions in host health. Annu. Rev. Food Sci. Technol. 7, 313–333. doi: 10.1146/annurev-food-041715-033159, PMID: 26772409

[B78] Kedia S. Virmani S. K Vuyyuru S. Kumar P. Kante B. Sahu P. . (2022). Faecal microbiota transplantation with anti-inflammatory diet (FMT-AID) followed by anti-inflammatory diet alone is effective in inducing and maintaining remission over 1 year in mild to moderate ulcerative colitis: a randomised controlled trial. Gut 71, 12. doi: 10.1136/gutjnl-2022-327811, PMID: 35973787

[B79] Kerry R. G. Patra J. K. Gouda S. Park Y. Shin H. S. Das G. (2018). Benefaction of probiotics for human health: A review. J. Food Drug Anal. 26, 3. doi: 10.1016/j.jfda.2018.01.002, PMID: 29976412 PMC9303019

[B80] Kirchberger S. Royston D. J. Boulard O. Thornton E. Franchini F. Szabady R. L. . (2013). Innate lymphoid cells sustain colon cancer through production of interleukin-22 in a mouse model. J. Exp. Med. 210, 5. doi: 10.1084/jem.20122308, PMID: 23589566 PMC3646494

[B81] Kobayashi M. Saitoh S. Tanimura N. Takahashi K. Kawasaki K. Nishijima M. . (2006). Regulatory roles for MD-2 and TLR4 in ligand-induced receptor clustering. J. Immunol. 176, 10. doi: 10.4049/jimmunol.176.10.6211, PMID: 16670331

[B82] Koh A. De Vadder F. Kovatcheva-Datchary P. Bäckhed F. (2016). From dietary fiber to host physiology: short-chain fatty acids as key bacterial metabolites. Cell 165, 6. doi: 10.1016/j.cell.2016.05.041, PMID: 27259147

[B83] Kostic A. D. Chun E. Robertson L. Glickman J. N. Gallini C. A. Michaud M. . (2013). Fusobacterium nucleatum potentiates intestinal tumorigenesis and modulates the tumor-immune microenvironment. Cell Host Microbe 14, 2. doi: 10.1016/j.chom.2013.07.007, PMID: 23954159 PMC3772512

[B84] Kumar R. Sood U. Gupta V. Singh M. Scaria J. Lal R. (2020). Recent advancements in the development of modern probiotics for restoring human gut microbiome dysbiosis. Indian J. Microbiol. 60, 1. doi: 10.1007/s12088-019-00808-y, PMID: 32089570 PMC7000592

[B85] Kwong T. N. Y. Wang X. Nakatsu G. Chow T. C. Tipoe T. Dai R. Z. W. . (2018). Association between bacteremia from specific microbes and subsequent diagnosis of colorectal cancer. Gastroenterology 155, 2. doi: 10.1053/j.gastro.2018.04.028, PMID: 29729257

[B86] Le B. Yang S. H. (2018). Efficacy of Lactobacillus plantarum in prevention of inflammatory bowel disease. Toxicol. Rep. 5, 314–317. doi: 10.1016/j.toxrep.2018.02.007, PMID: 29854599 PMC5977373

[B87] Lee J. G. Lee J. Lee A. R. Jo S. V. Park C. H. Han D. S. . (2022). Impact of short-chain fatty acid supplementation on gut inflammation and microbiota composition in a murine colitis model. J. Nutr. Biochem. 101, 3. doi: 10.1016/j.jnutbio.2021.108926, PMID: 34848335

[B88] Lei F. Zeng F. Yu X. Deng Y. Zhang Z. Xu M. . (2023). Oral hydrogel nanoemulsion co-delivery system treats inflammatory bowel disease via anti-inflammatory and promoting intestinal mucosa repair. J. Nanobiotechnol. 21, 1. doi: 10.1186/s12951-023-02045-4, PMID: 37596598 PMC10436423

[B89] Li H. Li L. Mei H. Pan G. Wang X. Huang X. . (2020a). Antitumor properties of triptolide: phenotype regulation of macrophage differentiation. Cancer Biol. Ther. 21, 2. doi: 10.1080/15384047.2019.1679555, PMID: 31663424 PMC7012063

[B90] Li M. van Esch B. Wagenaar G. T. M. Garssen J. Folkerts G. Henricks P. A. J. (2018). Pro- and anti-inflammatory effects of short chain fatty acids on immune and endothelial cells. Eur. J. Pharmacol. 831, 14. doi: 10.1016/j.ejphar.2018.05.003, PMID: 29750914

[B91] Li Q. Ding C. Meng T. Lu W. Liu W. Hao H. . (2017). Butyrate suppresses motility of colorectal cancer cells via deactivating Akt/ERK signaling in histone deacetylase dependent manner. J. Pharmacol. Sci. 135, 4. doi: 10.1016/j.jphs.2017.11.004, PMID: 29233468

[B92] Li Y. Li Q. Yuan R. Wang Y. Guo C. Wang L. (2024). Bifidobacterium breve-derived indole-3-lactic acid ameliorates colitis-associated tumorigenesis by directing the differentiation of immature colonic macrophages. Theranostics 14, 7. doi: 10.7150/thno.92350, PMID: 38773969 PMC11103503

[B93] Li H. Xing X. Zhang X. Li L. Jiang Z. Wang T. . (2020b). Effects of triptolide on the sphingosine kinase - Sphingosine-1-phosphate signaling pathway in colitis-associated colon cancer. Int. Immunopharmacol. 88, 11. doi: 10.1016/j.intimp.2020.106892, PMID: 32810834

[B94] Liu H. Dasgupta S. Fu Y. Bailey B. Roy C. Lightcap E. . (2019). Subsets of mononuclear phagocytes are enriched in the inflamed colons of patients with IBD. BMC Immunol. 20, 1. doi: 10.1186/s12865-019-0322-z, PMID: 31718550 PMC6852755

[B95] Liu H. Y. Gu F. Zhu C. Yuan L. Zhu C. Zhu M. . (2022). Epithelial heat shock proteins mediate the protective effects of limosilactobacillus reuteri in dextran sulfate sodium-induced colitis. Front. Immunol. 13. doi: 10.3389/fimmu.2022.865982, PMID: 35320932 PMC8934773

[B96] Liu L. Dong W. Wang S. Zhang Y. Liu T. Xie R. . (2018). Deoxycholic acid disrupts the intestinal mucosal barrier and promotes intestinal tumorigenesis. Food Funct. 9, 11. doi: 10.1039/c8fo01143e, PMID: 30339173

[B97] Liu L. Liang L. Liang H. Wang M. Lu B. Xue M. . (2019). Fusobacterium nucleatum Aggravates the Progression of Colitis by Regulating M1 Macrophage Polarization via AKT2 Pathway. Front. Immunol. 10. doi: 10.3389/fimmu.2019.01324, PMID: 31249571 PMC6582778

[B98] Liu L. Stokes J. V. Tan W. Pruett S. B. (2022). An optimized flow cytometry panel for classifying macrophage polarization. J. Immunol. Methods 511, 12. doi: 10.1016/j.jim.2022.113378, PMID: 36265578

[B99] Lloyd-Price J. Arze C. Ananthakrishnan A. N. Schirmer M. Avila-Pacheco J. Poon T. W. . (2019). Multi-omics of the gut microbial ecosystem in inflammatory bowel diseases. Nature 569, 7758. doi: 10.1038/s41586-019-1237-9, PMID: 31142855 PMC6650278

[B100] LoCascio R. G. Ninonuevo M. R. Freeman S. L. Sela D. A. Grimm R. Lebrilla C. B. . (2007). Glycoprofiling of bifidobacterial consumption of human milk oligosaccharides demonstrates strain specific, preferential consumption of small chain glycans secreted in early human lactation. J. Agric. Food Chem. 55, 22. doi: 10.1021/jf0710480, PMID: 17915960

[B101] Lu M. J. Qiu X. Y. Mao X. Q. Li X. T. Zhang H. J. (2018). Systematic review with meta-analysis: thiopurines decrease the risk of colorectal neoplasia in patients with inflammatory bowel disease. Aliment. Pharmacol. Ther. 47, 3. doi: 10.1111/apt.14436, PMID: 29205426

[B102] Luo M. Du Y. Liu X. Zhang S. Zhu W. Liu K. . (2025). Fecal microbiota transplantation alleviates cirrhotic portal hypertension in rats via butyrate-mediated HDAC3 inhibition and PI3K/Akt/eNOS signaling regulation. Eur. J. Pharmacol. 1002, 17. doi: 10.1016/j.ejphar.2025.177781, PMID: 40441587

[B103] Luo M. Zhao F. Cheng H. Su M. Wang Y. (2024). Macrophage polarization: an important role in inflammatory diseases. Front. Immunol. 15. doi: 10.3389/fimmu.2024.1352946, PMID: 38660308 PMC11039887

[B104] Luyendyk J. P. Schabbauer G. A. Tencati M. Holscher T. Pawlinski R. Mackman N. (2008). Genetic analysis of the role of the PI3K-Akt pathway in lipopolysaccharide-induced cytokine and tissue factor gene expression in monocytes/macrophages. J. Immunol. 180, 6. doi: 10.4049/jimmunol.180.6.4218, PMID: 18322234 PMC2834303

[B105] MacPherson C. W. Shastri P. Mathieu O. Tompkins T. A. Burguière P. (2017). Genome-wide immune modulation of TLR3-mediated inflammation in intestinal epithelial cells differs between single and multi-strain probiotic combination. PloS One 12, 1. doi: 10.1371/journal.pone.0169847, PMID: 28099447 PMC5242491

[B106] Mańkowska-Wierzbicka D. Stelmach-Mardas M. Gabryel M. Tomczak H. Skrzypczak-Zielińska M. Zakerska-Banaszak O. . (2020). The effectiveness of multi-session FMT treatment in active ulcerative colitis patients: A pilot study. Biomedicines 8, 8. doi: 10.3390/biomedicines8080268, PMID: 32756350 PMC7459721

[B107] Marelli G. Erreni M. Anselmo A. Taverniti V. Guglielmetti S. Mantovani A. . (2017). Heme-oxygenase-1 production by intestinal CX3CR1(+) macrophages helps to resolve inflammation and prevents carcinogenesis. Cancer Res. 77, 16. doi: 10.1158/0008-5472.Can-16-2501, PMID: 28619710

[B108] Martinez F. O. Gordon S. (2014). The M1 and M2 paradigm of macrophage activation: time for reassessment. F1000Prime. Rep. 6, 13. doi: 10.12703/p6-13, PMID: 24669294 PMC3944738

[B109] McLean M. H. Murray G. I. Stewart K. N. Norrie G. Mayer C. Hold G. L. . (2011). The inflammatory microenvironment in colorectal neoplasia. PloS One 6, 1. doi: 10.1371/journal.pone.0015366, PMID: 21249124 PMC3017541

[B110] Meng D. Sommella E. Salviati E. Campiglia P. Ganguli K. Djebali K. . (2020). Indole-3-lactic acid, a metabolite of tryptophan, secreted by Bifidobacterium longum subspecies infantis is anti-inflammatory in the immature intestine. Pediatr. Res. 88, 2. doi: 10.1038/s41390-019-0740-x, PMID: 31945773 PMC7363505

[B111] Müller S. Kohanbash G. Liu S. J. Alvarado B. Carrera D. Bhaduri A. . (2017). Single-cell profiling of human gliomas reveals macrophage ontogeny as a basis for regional differences in macrophage activation in the tumor microenvironment. Genome Biol. 18, 1. doi: 10.1186/s13059-017-1362-4, PMID: 29262845 PMC5738907

[B112] Murray I. A. Patterson A. D. Perdew G. H. (2014). Aryl hydrocarbon receptor ligands in cancer: friend and foe. Nat. Rev. Cancer. 14, 12. doi: 10.1038/nrc3846, PMID: 25568920 PMC4401080

[B113] Murray P. J. Wynn T. A. (2011). Protective and pathogenic functions of macrophage subsets. Nat. Rev. Immunol. 11, 11. doi: 10.1038/nri3073, PMID: 21997792 PMC3422549

[B114] Nagai Y. Akashi S. Nagafuku M. Ogata M. Iwakura Y. Akira S. . (2002). Essential role of MD-2 in LPS responsiveness and TLR4 distribution. Nat. Immunol. 3, 7. doi: 10.1038/ni809, PMID: 12055629

[B115] Natoli M. Herzig P. Pishali Bejestani E. Buchi M. Ritschard R. Lloyd G. K. . (2021). Plinabulin, a distinct microtubule-targeting chemotherapy, promotes M1-like macrophage polarization and anti-tumor immunity. Front. Oncol. 11. doi: 10.3389/fonc.2021.644608, PMID: 33747968 PMC7966525

[B116] O’Callaghan A. van Sinderen D. (2016). Bifidobacteria and their role as members of the human gut microbiota. Front. Microbiol. 7. doi: 10.3389/fmicb.2016.00925, PMID: 27379055 PMC4908950

[B117] Ocvirk S. O’Keefe S. J. (2017). Influence of bile acids on colorectal cancer risk: potential mechanisms mediated by diet - gut microbiota interactions. Curr. Nutr. Rep. 6, 4. doi: 10.1007/s13668-017-0219-5, PMID: 29430336 PMC5802424

[B118] Odegaard J. I. Chawla A. (2011). Alternative macrophage activation and metabolism. Annu. Rev. Pathol. 6, 275–297. doi: 10.1146/annurev-pathol-011110-130138, PMID: 21034223 PMC3381938

[B119] Orecchioni M. Ghosheh Y. Pramod A. B. Ley K. (2019). Macrophage Polarization: Different Gene Signatures in M1(LPS+) vs. Classically and M2(LPS-) vs. Alternatively Activated Macrophages. Front. Immunol. 10. doi: 10.3389/fimmu.2019.01084, PMID: 31178859 PMC6543837

[B120] Papalexi E. Satija R. (2018). Single-cell RNA sequencing to explore immune cell heterogeneity. Nat. Rev. Immunol. 18, 1. doi: 10.1038/nri.2017.76, PMID: 28787399

[B121] Petrulis J. R. Perdew G. H. (2002). The role of chaperone proteins in the aryl hydrocarbon receptor core complex. Chem. Biol. Interact. 141, 1–2. doi: 10.1016/s0009-2797(02)00064-9, PMID: 12213383

[B122] Qiu J. Heller J. J. Guo X. Chen Z. M. Fish K. Fu Y. X. . (2012). The aryl hydrocarbon receptor regulates gut immunity through modulation of innate lymphoid cells. Immunity 36, 1. doi: 10.1016/j.immuni.2011.11.011, PMID: 22177117 PMC3268875

[B123] Qiu P. Ishimoto T. Fu L. Zhang J. Zhang Z. Liu Y. (2022). The gut microbiota in inflammatory bowel disease. Front. Cell Infect. Microbiol. 12. doi: 10.3389/fcimb.2022.733992, PMID: 35273921 PMC8902753

[B124] Rao X. Zhou X. Wang G. Jie X. Xing B. Xu Y. . (2022). NLRP6 is required for cancer-derived exosome-modified macrophage M2 polarization and promotes metastasis in small cell lung cancer. Cell Death Dis. 13, 10. doi: 10.1038/s41419-022-05336-0, PMID: 36270983 PMC9587220

[B125] Ratajczak W. Rył A. Mizerski A. Walczakiewicz K. Sipak O. Laszczyńska M. (2019). Immunomodulatory potential of gut microbiome-derived short-chain fatty acids (SCFAs). Acta Biochim. Pol. 66, 1. doi: 10.18388/abp.2018_2648, PMID: 30831575

[B126] Ren K. Yong C. Jin Y. Rong S. Xue K. Cao B. . (2025). Unraveling the microbial mysteries: gut microbiota’s role in ulcerative colitis. Front. Nutr. 12. doi: 10.3389/fnut.2025.1519974, PMID: 39996003 PMC11847676

[B127] Ridlon J. M. Kang D. J. Hylemon P. B. (2006). Bile salt biotransformations by human intestinal bacteria. J. Lipid Res. 47, 2. doi: 10.1194/jlr.R500013-JLR200, PMID: 16299351

[B128] Rossol M. Heine H. Meusch U. Quandt D. Klein C. Sweet M. J. . (2011). LPS-induced cytokine production in human monocytes and macrophages. Crit. Rev. Immunol. 31, 5. doi: 10.1615/critrevimmunol.v31.i5.20, PMID: 22142165

[B129] Rothhammer V. Mascanfroni I. D. Bunse L. Takenaka M. C. Kenison J. E. Mayo L. . (2016). Type I interferons and microbial metabolites of tryptophan modulate astrocyte activity and central nervous system inflammation via the aryl hydrocarbon receptor. Nat. Med. 22, 6. doi: 10.1038/nm.4106, PMID: 27158906 PMC4899206

[B130] Sakai K. De Velasco M. A. Kura Y. Nishio K. (2021). Transcriptome profiling and metagenomic analysis help to elucidate interactions in an inflammation-associated cancer mouse model. Cancers (Basel). 13, 15. doi: 10.3390/cancers13153683, PMID: 34359585 PMC8345192

[B131] Sato N. Yuzawa M. Aminul M. I. Tomokiyo M. Albarracin L. Garcia-Castillo V. . (2021). Evaluation of porcine intestinal epitheliocytes as an *in vitro* immunoassay system for the selection of probiotic bifidobacteria to alleviate inflammatory bowel disease. Probiot. Antimicrob. Proteins. 13, 3. doi: 10.1007/s12602-020-09694-z, PMID: 32779098

[B132] Schmid M. C. Avraamides C. J. Dippold H. C. Franco I. Foubert P. Ellies L. G. . (2011). Receptor tyrosine kinases and TLR/IL1Rs unexpectedly activate myeloid cell PI3kγ, a single convergent point promoting tumor inflammation and progression. Cancer Cell. 19, 6. doi: 10.1016/j.ccr.2011.04.016, PMID: 21665146 PMC3144144

[B133] Schoeler M. Caesar R. (2019). Dietary lipids, gut microbiota and lipid metabolism. Rev. Endocr. Metab. Disord. 20, 4. doi: 10.1007/s11154-019-09512-0, PMID: 31707624 PMC6938793

[B134] Scott N. A. Andrusaite A. Andersen P. Lawson M. Alcon-Giner C. Leclaire C. . (2018). Antibiotics induce sustained dysregulation of intestinal T cell immunity by perturbing macrophage homeostasis. Sci. Transl. Med. 10, 464. doi: 10.1126/scitranslmed.aao4755, PMID: 30355800 PMC6548564

[B135] Scott S. A. Fu J. Chang P. V. (2020). Microbial tryptophan metabolites regulate gut barrier function via the aryl hydrocarbon receptor. Proc. Natl. Acad. Sci. U. S. A. 117, 32. doi: 10.1073/pnas.2000047117, PMID: 32719140 PMC7431026

[B136] Shao Y. Evers S. S. Shin J. H. Ramakrishnan S. K. Bozadjieva-Kramer N. Yao Q. . (2022). Vertical sleeve gastrectomy increases duodenal Lactobacillus spp. richness associated with the activation of intestinal HIF2α signaling and metabolic benefits. Mol. Metab. 57, 3. doi: 10.1016/j.molmet.2022.101432, PMID: 34998940 PMC8790500

[B137] Shao X. Sun S. Zhou Y. Wang H. Yu Y. Hu T. . (2021). Bacteroides fragilis restricts colitis-associated cancer via negative regulation of the NLRP3 axis. Cancer Lett. 523, 28. doi: 10.1016/j.canlet.2021.10.002, PMID: 34627951

[B138] Sica A. Mantovani A. (2012). Macrophage plasticity and polarization: *in vivo* veritas. J. Clin. Invest. 122, 3. doi: 10.1172/jci59643, PMID: 22378047 PMC3287223

[B139] Singh S. Bhatia R. Khare P. Sharma S. Rajarammohan S. Bishnoi M. . (2020). Anti-inflammatory Bifidobacterium strains prevent dextran sodium sulfate induced colitis and associated gut microbial dysbiosis in mice. Sci. Rep. 10, 1. doi: 10.1038/s41598-020-75702-5, PMID: 33122795 PMC7596498

[B140] Song Q. Gao Y. Liu K. Tang Y. Man Y. Wu H. (2024). Gut microbial and metabolomics profiles reveal the potential mechanism of fecal microbiota transplantation in modulating the progression of colitis-associated colorectal cancer in mice. J Transl Med 22, 1. doi: 10.1186/s12967-024-05786-4, PMID: 39548468 PMC11566892

[B141] Sonnenburg J. L. Bäckhed F. (2016). Diet-microbiota interactions as moderators of human metabolism. Nature 535, 7610. doi: 10.1038/nature18846, PMID: 27383980 PMC5991619

[B142] Steinbach E. C. Plevy S. E. (2014). The role of macrophages and dendritic cells in the initiation of inflammation in IBD. Inflammation Bowel. Dis. 20, 1. doi: 10.1097/MIB.0b013e3182a69dca, PMID: 23974993 PMC4098861

[B143] Su X. Gao Y. Yang R. (2023). Gut microbiota derived bile acid metabolites maintain the homeostasis of gut and systemic immunity. Front. Immunol. 14. doi: 10.3389/fimmu.2023.1127743, PMID: 37256134 PMC10225537

[B144] Sui H. Tan H. Fu J. Song Q. Jia R. Han L. . (2020). The active fraction of Garcinia yunnanensis suppresses the progression of colorectal carcinoma by interfering with tumorassociated macrophage-associated M2 macrophage polarization *in vivo* and *in vitro*. FASEB J. 34, 6. doi: 10.1096/fj.201903011R, PMID: 32283574

[B145] Tang Y. Feng X. Lu Q. Cui C. Yu M. Wen Z. . (2024). MZB1-mediated IgA secretion suppresses the development and progression of colorectal cancer triggered by gut inflammation. Mucosal Immunol. 17, 3. doi: 10.1016/j.mucimm.2023.12.002, PMID: 38101774

[B146] Tang L. Zhang H. Wang C. Li H. Zhang Q. Bai J. (2017). M2A and M2C macrophage subsets ameliorate inflammation and fibroproliferation in acute lung injury through interleukin 10 pathway. Shock 48, 1. doi: 10.1097/shk.0000000000000820, PMID: 27941591

[B147] Usami M. Kishimoto K. Ohata A. Miyoshi M. Aoyama M. Fueda Y. . (2008). Butyrate and trichostatin A attenuate nuclear factor kappaB activation and tumor necrosis factor alpha secretion and increase prostaglandin E2 secretion in human peripheral blood mononuclear cells. Nutr. Res. 28, 5. doi: 10.1016/j.nutres.2008.02.012, PMID: 19083427

[B148] Vernia F. Longo S. Stefanelli G. Viscido A. Latella G. (2021). Dietary factors modulating colorectal carcinogenesis. Nutrients 13, 1. doi: 10.3390/nu13010143, PMID: 33401525 PMC7824178

[B149] Verreck F. A. de Boer T. Langenberg D. M. Hoeve M. A. Kramer M. Vaisberg E. . (2004). Human IL-23-producing type 1 macrophages promote but IL-10-producing type 2 macrophages subvert immunity to (myco)bacteria. Proc. Natl. Acad. Sci. U. S. A. 101, 13. doi: 10.1073/pnas.0400983101, PMID: 15070757 PMC384786

[B150] Vich Vila A. Imhann F. Collij V. Jankipersadsing S. A. Gurry T. Mujagic Z. . (2018). Gut microbiota composition and functional changes in inflammatory bowel disease and irritable bowel syndrome. Sci. Transl. Med. 10, 472. doi: 10.1126/scitranslmed.aap8914, PMID: 30567928

[B151] Wang J. W. Pan Y. B. Cao Y. Q. Wang C. Jiang W. D. Zhai W. F. . (2020). Loganin alleviates LPS-activated intestinal epithelial inflammation by regulating TLR4/NF-κB and JAK/STAT3 signaling pathways. Kaohsiung. J. Med. Sci. 36, 4. doi: 10.1002/kjm2.12160, PMID: 31859422 PMC11896463

[B152] Wang L. Gong Z. Zhang X. Zhu F. Liu Y. Jin C. . (2020). Gut microbial bile acid metabolite skews macrophage polarization and contributes to high-fat diet-induced colonic inflammation. Gut. Microbes 12, 1. doi: 10.1080/19490976.2020.1819155, PMID: 33006494 PMC7553752

[B153] Wang R. Cao S. Bashir M. E. H. Hesser L. A. Su Y. Hong S. M. C. . (2023). Treatment of peanut allergy and colitis in mice via the intestinal release of butyrate from polymeric micelles. Nat. BioMed. Eng. 7, 1. doi: 10.1038/s41551-022-00972-5, PMID: 36550307 PMC9870785

[B154] Wang S. Liu R. Yu Q. Dong L. Bi Y. Liu G. (2019). Metabolic reprogramming of macrophages during infections and cancer. Cancer Lett. 452, 12. doi: 10.1016/j.canlet.2019.03.015, PMID: 30905817

[B155] Wang Y. Fang Z. Zhai Q. Cui S. Zhao J. Zhang H. . (2021). Supernatants of Bifidobacterium longum and Lactobacillus plantarum Strains Exhibited Antioxidative Effects on A7R5 Cells. Microorganisms 9, 2. doi: 10.3390/microorganisms9020452, PMID: 33671556 PMC7927071

[B156] Wang Y. Wiesnoski D. H. Helmink B. A. Gopalakrishnan V. Choi K. DuPont H. L. . (2018). Fecal microbiota transplantation for refractory immune checkpoint inhibitor-associated colitis. Nat. Med. 24, 12. doi: 10.1038/s41591-018-0238-9, PMID: 30420754 PMC6322556

[B157] Wardill H. R. Secombe K. R. Bryant R. V. Hazenberg M. D. Costello S. P. (2019). Adjunctive fecal microbiota transplantation in supportive oncology: Emerging indications and considerations in immunocompromised patients. EBioMedicine 44, 6. doi: 10.1016/j.ebiom.2019.03.070, PMID: 30940601 PMC6603490

[B158] Whittle B. J. Varga C. (2010). New light on the anti-colitic actions of therapeutic aminosalicylates: the role of heme oxygenase. Pharmacol. Rep. 62, 3. doi: 10.1016/s1734-1140(10)70312-1, PMID: 20631420

[B159] Winter S. E. Winter M. G. Xavier M. N. Thiennimitr P. Poon V. Keestra A. M. . (2013). Host-derived nitrate boosts growth of E. coli in the inflamed gut. Science 339, 6120. doi: 10.1126/science.1232467, PMID: 23393266 PMC4004111

[B160] Wu S. Rhee K. J. Albesiano E. Rabizadeh S. Wu X. Yen H. R. . (2009). A human colonic commensal promotes colon tumorigenesis via activation of T helper type 17 T cell responses. Nat. Med. 15, 9. doi: 10.1038/nm.2015, PMID: 19701202 PMC3034219

[B161] Wu Y. Yao J. Xie J. Liu Z. Zhou Y. Pan H. . (2018). The role of autophagy in colitis-associated colorectal cancer. Signal Transduct. Target. Ther. 3, 31. doi: 10.1038/s41392-018-0031-8, PMID: 30510778 PMC6265276

[B162] Wu K. Yuan Y. Yu H. Dai X. Wang S. Sun Z. . (2020). The gut microbial metabolite trimethylamine N-oxide aggravates GVHD by inducing M1 macrophage polarization in mice. Blood 136, 4. doi: 10.1182/blood.2019003990, PMID: 32291445 PMC7378459

[B163] Xu H. Hiraishi K. Kurahara L. H. Nakano-Narusawa Y. Li X. Hu Y. . (2021). Inhibitory effects of breast milk-derived lactobacillus rhamnosus probio-M9 on colitis-associated carcinogenesis by restoration of the gut microbiota in a mouse model. Nutrients 13, 4. doi: 10.3390/nu13041143, PMID: 33808480 PMC8065529

[B164] Xu Y. Cai Q. Zhao C. Zhang W. Xu X. Lin H. . (2024). Gegen qinlian decoction attenuates colitis-associated colorectal cancer via suppressing TLR4 signaling pathway based on network pharmacology and *in vivo*/*in* vitro experimental validation. Pharm. (Basel). 18, 1. doi: 10.3390/ph18010012, PMID: 39861077 PMC11768880

[B165] Xun J. Zhou S. Lv Z. Wang B. Luo H. Zhang L. . (2023). Dioscin modulates macrophages polarization and MDSCs differentiation to inhibit tumorigenesis of colitis-associated colorectal cancer. Int. Immunopharmacol. 117, 4. doi: 10.1016/j.intimp.2023.109839, PMID: 36809720

[B166] Yan Y. Tian L. Zhao Y. Xuan B. Xu X. Ding J. . (2025). Bacteroides fragilis Toxin Suppresses METTL3-Mediated m6A Modification in Macrophage to Promote Inflammatory Bowel Disease. J. Crohns. Colitis. 19, 3. doi: 10.1093/ecco-jcc/jjae179, PMID: 40065724

[B167] Yang Y. Li L. Xu C. Wang Y. Wang Z. Chen M. . (2020). Cross-talk between the gut microbiota and monocyte-like macrophages mediates an inflammatory response to promote colitis-associated tumourigenesis. Gut 70, 8. doi: 10.1136/gutjnl-2020-320777, PMID: 33122176 PMC8292576

[B168] Yang W. Yu T. Huang X. Bilotta A. J. Xu L. Lu Y. . (2020). Intestinal microbiota-derived short-chain fatty acids regulation of immune cell IL-22 production and gut immunity. Nat. Commun. 11, 1. doi: 10.1038/s41467-020-18262-6, PMID: 32901017 PMC7478978

[B169] Yao S. Zhao Z. Wang W. Liu X. (2021). Bifidobacterium longum: protection against inflammatory bowel disease. J. Immunol. Res. 2021, 8030297. doi: 10.1155/2021/8030297, PMID: 34337079 PMC8324359

[B170] Yin Y. Wan J. Yu J. Wu K. (2023). Molecular pathogenesis of colitis-associated colorectal cancer: immunity, genetics, and intestinal microecology. Inflammation Bowel. Dis. 29, 10. doi: 10.1093/ibd/izad081, PMID: 37202830

[B171] Yu K. Li Q. Sun X. Peng X. Tang Q. Chu H. . (2023). Bacterial indole-3-lactic acid affects epithelium-macrophage crosstalk to regulate intestinal homeostasis. Proc. Natl. Acad. Sci. U. S. A. 120, 45. doi: 10.1073/pnas.2309032120, PMID: 37903267 PMC10636326

[B172] Yuan Q. Gu J. Zhang J. Liu S. Wang Q. Tian T. . (2021). MyD88 in myofibroblasts enhances colitis-associated tumorigenesis via promoting macrophage M2 polarization. Cell Rep. 34, 5. doi: 10.1016/j.celrep.2021.108724, PMID: 33535045

[B173] Yunna C. Mengru H. Lei W. Weidong C. (2020). Macrophage M1/M2 polarization. Eur. J. Pharmacol. 877, 12. doi: 10.1016/j.ejphar.2020.173090, PMID: 32234529

[B174] Zhang W. Chen L. Ma K. Zhao Y. Liu X. Wang Y. . (2016). Polarization of macrophages in the tumor microenvironment is influenced by EGFR signaling within colon cancer cells. Oncotarget 7, 46. doi: 10.18632/oncotarget.12207, PMID: 27683110 PMC5342747

[B175] Zhang L. Li Z. Skrzypczynska K. M. Fang Q. Zhang W. O’Brien S. A. . (2020). Single-cell analyses inform mechanisms of myeloid-targeted therapies in colon cancer. Cell 181, 2. doi: 10.1016/j.cell.2020.03.048, PMID: 32302573

[B176] Zhang M. Li X. Zhang Q. Yang J. Liu G. (2023). Roles of macrophages on ulcerative colitis and colitis-associated colorectal cancer. Front. Immunol. 14. doi: 10.3389/fimmu.2023.1103617, PMID: 37006260 PMC10062481

[B177] Zhang B. Pan H. Chen Z. Yin T. Zheng M. Cai L. (2023). Twin-bioengine self-adaptive micro/nanorobots using enzyme actuation and macrophage relay for gastrointestinal inflammation therapy. Sci. Adv. 9, 8. doi: 10.1126/sciadv.adc8978, PMID: 36812317 PMC9946363

[B178] Zhang L. Zhang Z. (2019). Recharacterizing tumor-infiltrating lymphocytes by single-cell RNA sequencing. Cancer Immunol. Res. 7, 7. doi: 10.1158/2326-6066.Cir-18-0658, PMID: 31262773

[B179] Zhang Q. Zhao Q. Li T. Lu L. Wang F. Zhang H. . (2023). Lactobacillus plantarum-derived indole-3-lactic acid ameliorates colorectal tumorigenesis via epigenetic regulation of CD8(+) T cell immunity. Cell Metab. 35, 6. doi: 10.1016/j.cmet.2023.04.015, PMID: 37192617

[B180] Zhao S. Gong Z. Zhou J. Tian C. Gao Y. Xu C. . (2016). Deoxycholic acid triggers NLRP3 inflammasome activation and aggravates DSS-induced colitis in mice. Front. Immunol. 7. doi: 10.3389/fimmu.2016.00536, PMID: 27965665 PMC5124666

[B181] Zhao Y. Jiang Q. (2021). Roles of the polyphenol-gut microbiota interaction in alleviating colitis and preventing colitis-associated colorectal cancer. Adv. Nutr. 12, 2. doi: 10.1093/advances/nmaa104, PMID: 32905583 PMC8009754

[B182] Zheng Y. Valdez P. A. Danilenko D. M. Hu Y. Sa S. M. Gong Q. . (2008). Interleukin-22 mediates early host defense against attaching and effacing bacterial pathogens. Nat. Med. 14, 3. doi: 10.1038/nm1720, PMID: 18264109

